# Standard Elements in Studies of Adverse Events and Medical Error (SESAME): explanation and elaboration

**DOI:** 10.1097/pq9.0000000000000930

**Published:** 2026-09-11

**Authors:** Richard T. Griffey, Lee M. Adler, David C. Stockwell, Peter D. Hibbert, Dorthe O. Klein, Rene Schwendimann, Andrew D. Auerbach, Rachel Ancona, Ryan M. Schneider, David Classen, Christopher R. Carpenter, Anne W S. Rutjes, Maria Unbeck, The SESAME Development Team, Lee M Adler

**Affiliations:** 1 Department of Emergency Medicine, Washington University in St. Louis School of Medicine, St. Louis, Missouri, USA; 2 Clinical Quality and Safety, AdventHealth, Altamonte Springs, Florida, USA; 3 Anesthesiology and Critical Care Medicine, Johns Hopkins University, Baltimore, Maryland, USA; 4 Australian Institute of Health Innovation, Macquarie University, Sydney, New South Wales, Australia; 5 Clinical Epidemiology and Medical Technology Assessment, Maastricht University Medical Centre+, Maastricht, The Netherlands; 6 Care and Public Health Research Insititute, Maastricht University, Maastricht, The Netherlands; 7 Institute of Nursing Science, University of Basel, Basel, Switzerland; 8 Internal Medicine, University of California, San Francisco, San Francisco, California, USA; 9 Division of Epidemiology, University of Utah, Salt Lake City, Utah, USA; 10 PascalMetrics, Washington, DC, USA; 11 Emergency Medicine, Mayo Clinic, Rochester, Minnesota, USA; 12 Departmental Faculty of Medicine, UniCamillus - Saint Camillus International University of Health and Medical Science, Rome, Lazio, Italy; 13 School of Health and Welfare, Dalarna University, Falun, Sweden; 14 Department of Clinical Sciences, Danderyd Hospital, Karolinska Institutet, Stockholm, Sweden

## Abstract

Studies that evaluate adverse events and medical error often suffer from incomplete and inadequate reporting, which affects the interpretability and reproducibility of their findings. To address this, we developed the 44-item Standard Elements in Studies of Adverse Event or Medical Error (SESAME) checklist using a rigorous process following the recommendations of the Enhancing the QUAlity and Transparency Of health Research Network. The aim of this manuscript is to explain the scope and aim of the checklist by providing a detailed explanation of each item in the SESAME checklist, illustrated by examples from selected studies of adverse events and medical error.

## INTRODUCTION

At the time of this writing, there are over 500 reporting guidelines indexed in the Enhancing the QUAlity and Transparency Of health Research (EQUATOR) Network.^1^ These help with methodological reporting standards for a broad range of study types. However, despite being an area with a depth of study, to date, there is not a general reporting guideline that addresses research on adverse events (AEs) in healthcare and medical error. AEs are reported as incidental outcomes or side effects of procedures, medications or clinical trials and there are in fact reporting guideline extensions to address these kinds of events. However, for studies where the primary outcome relates to AEs or medical error and/or the measurement or frequency of such events, guidance is missing. Multiple systematic reviews have highlighted variability in reporting within this field of research, with consequent challenges in comparing studies, AE rates and developing a common understanding of findings and lessons learnt.^2,3^

This led to the development of the Standard Elements in Studies of Adverse Event or Medical Error (SESAME) guideline (www.sesamestatement.org).www.sesamestatement.org^3^ Following established guidelines, we convened a steering committee and recruited an international development group. In a series of web-based meetings, we iteratively crafted the checklist, using a modified Delphi process to vote on inclusion and wording of items, adhering to the ACcurate COnsensus Reporting Document criteria.^4^ The full project lifecycle, from initiation to completion, spanned a duration of 2 years. The development of the guideline is described in a separate manuscript.^3^ Here, we present the content of the checklist in detail and explain how to use it.

Like several existing checklists, SESAME is not limited to a single research methodology. It is designed for broad applicability across diverse study designs and can be used in conjunction with other guidelines that focus primarily on a specific methodology. Although emerging approaches include computerised surveillance and artificial intelligence for the detection and prevention of AEs, retrospective record review remains the gold standard in research on AEs and medical error, and the clinical judgement inherent in this process forms the foundation for other approaches including algorithm development used in electronic or prospective detection systems.

Since the publication of the Harvard Medical Practice Study (HMPS)^5^ and subsequent development of the Institute for Healthcare Improvement’s (IHI) Global Trigger Tool (GTT),^6^ there have been many publications observing different AE rates and different findings based on the approach used.^7–14^ The purpose of the SESAME guideline is not to advance a particular approach but rather to facilitate transparency by increasing the quality and completeness of reporting. The aim of this manuscript is to explain the scope and aim of the checklist by providing a detailed explanation of each item in the SESAME checklist, illustrated by examples from selected studies of AEs and medical error.

In the next sections, we explain how to apply the tool and provide a detailed explanation of each item included in the SESAME checklist along with examples from the medical literature.

## Elaboration on individual checklist items

**Table:** 

Title, abstract and keywords
1. Title: include a commonly used term (eg, adverse event, near-miss) that conveys the study topic in the title.
Examples 1. “Adverse Drug Events in Ambulatory Care: A Cross-Sectional Study”^15^ 2. “Near-Miss Events Detected Using the Emergency Department Trigger Tool”^16^ 3. “Preventable Adverse Events in Surgical Care in Sweden: A Nationwide Review of Patient Notes”^17^ 4. “Diagnostic Errors in Hospitalized Adults Who Died or Were Transferred to Intensive Care”^18^
Explanation In addition to the study design, it is important to include a commonly used term to convey the topic for research within the realm of patient safety. This might include terms such as harm, failure to rescue events or non-harm events. This will facilitate straightforward identification by readers, for example, when searching bibliographic databases.^19^

**Table:** 

2. Abstract: provide an informative and balanced summary of background, objectives, methods, results and conclusion.
Example “**OBJECTIVES:** Previous studies have revealed racial/ethnic and socioeconomic disparities in quality of care and patient safety. However, these disparities have not been examined in a pediatric inpatient environment by using a measure of clinically confirmed adverse events (AEs). In this study, we do so using the Global Assessment of Pediatric Patient Safety (GAPPS) Trigger Tool. **METHODS:** GAPPS was applied to medical records of randomly selected pediatric patients discharged from 16 hospitals in the Pediatric Research in Inpatient Settings Network across US regions from January 2007 to December 2012. Disparities in AEs for hospitalized children were identified on the basis of patient race/ethnicity (black, Latino, white, or other; N=17 336 patient days) and insurance status (public, private or self-pay/no insurance; N=19 030 patient days). **RESULTS:** Compared with hospitalized non-Latino white children, hospitalized Latino children experienced higher rates of all AEs (Latino: 30.1 AEs per 1000 patient days vs white: 16.9 AEs per 1000 patient days; P=0.001), preventable AEs (Latino: 15.9 AEs per 1000 patient days vs white: 8.9 AEs per 1000 patient days; P=0.002) and high-severity AEs (Latino: 12.6 AEs per 1000 patient days vs white: 7.7 AEs per 1000 patient days; P=0.02). Compared with privately insured children, publicly insured children experienced higher rates of preventable AEs (public: 12.1 AEs per 1000 patient days vs private: 8.5 AEs per 1000 patient days; p=0.02). No significant differences were observed among other groups. **CONCLUSIONS:** The GAPPS analysis revealed racial and/or ethnic and socioeconomic disparities in rates of AEs experienced by hospitalised children across a broad range of geographic and hospital settings. Further investigation may reveal underlying mechanisms of these disparities and could help hospitals reduce harm.”^20^
Explanation The abstract in the example describes the article’s main objectives, methods, results and conclusions. This allows readers to quickly grasp the core content without reading the full text. Journals and various manuscript types may have different formatting requirements and preferences for abstracts. A well-structured abstract increases the chances of the article being found by search engines and bibliographic databases, improving its visibility and impact.^19,21,22^

**Table:** 

3 Keywords: use informative wording, including keywords, to facilitate correct indexing and retrievability in bibliographic databases.
Example “Adverse event; medical error; patient safety; oncology; risk factors; epidemiology”^23^
Explanation Between 1980 and 2012, the output of published scientific evidence across fields increased 9% annually, which represented a doubling of discoverable research every decade. If scientific research is not discoverable via common electronic search engines like PubMed and EMBASE, this may blunt the impact of the work.^24^ Authors select keywords and database indexers select Medical Subject Headings (MeSH) or similar terms in an effort to more consistently categorise medical research. The use of appropriate keywords facilitates correct indexing and retrievability in bibliographic databases.^19,21^ If authors have uncertainty around the most appropriate keyword, consultation with a medical librarian may prove helpful.^22^ However, adverse event (AE) descriptions vary across studies, making indexing to a single concept more challenging. For example, one study might index the descriptor ‘nausea’ while another uses ‘GI upset’. In addition, MeSH terms for nuanced or rare events may not exist.^25^ Notably, several key terms frequently used in this research area, such as *AE*, *incident* and *no harm event,* are not indexed as MeSH terms.

**Table:** 

4. Introduction: background/ rationale—describe the scientific and clinical background and rationale for undertaking the study (eg, to characterise harm, measure the adverse event rate, test or compare a surveillance tool).
Examples 1. “The identification and reduction of adverse events (AEs) is of great importance in order to minimize patient suffering. There are several methods to identify AEs. Retrospective record review (RRR) is a commonly used method. It is considered less sensitive to under-reporting than other methods, such as clinical incident reports, patient safety indicators, complaints or medicolegal claims. RRR can be repeated over time, and specific AE types can be identified (…). Studies indicate that orthopedics is one of the specialties where AEs are most common. The national incidence and pattern of AEs in Swedish orthopedic care have never been described. Therefore, the aim of this study was to describe the incidence, nature, preventability and consequences of AEs in Swedish orthopedic care.”^26^ 2. “Previous studies have identified harm using a trigger tool to detect specific “triggers,” defined as medical record-based hints, presented in a patient’s medical record that may be associated with harm. In a multisite pediatric study, the rate of harm was reported as 40 harms per 100 admissions. These results are consistent with findings in pediatrics and adult medicine. To better understand the proportion of the total harm burden that HACs represent, we compared the number of CMS-defined HACs to the total harms identified using a previously published pediatric trigger tool.”^27^
Explanation The background section of a manuscript should frame the significance of the problem, cite limitations of existing evidence and link the rationale logically to the study’s objective. This should identify gaps in current understanding and an explicit description of what the study aims to address. In the example, this is achieved by the provision of an overview of existing knowledge on the topic, by identifying gaps in current understanding and by the explicit description of what the study aims to address.

**Table:** 

5. Introduction: objectives—state specific objective(s).
Examples 1. “The aim of this study was to explore the incidence, preventability and nature of AEs occurring in hip fracture patients up to 90 days after surgery.”^28^ 2. “The current study assessed changes in adverse event rates for patient safety between 2010 and 2019 for acute myocardial infarction, heart failure, pneumonia, and major surgical procedures (the 4 patient groups previously studied) and a fifth group encompassing all other conditions.”^29^ 3. “This study is designed to determine which of the events designated as harms or AEs, are currently 1) identifiable algorithmically, 2) which are identifiable using other methods, and 3) which are likely going undetected due to the methodology used.”^30^
Explanation Clear objectives help readers to understand the study’s scope and the questions it is attempting to address. The objectives explain the research questions or hypotheses, which then inform the choice of study design. A clear description of study objectives thus allows the reader to verify if the study methods chosen are adequate to answer the study objectives. In these examples, descriptions are concise, specifying the outcome measures of interest, the target conditions or populations of interest and the time window to measure the outcomes of interest.

**Table:** 

6a. Methods: study design—describe the study design (eg, retrospective or prospective; observational or interventional).
Examples 1. “This study used a retrospective record review design (…).”^31^ 2. “We conducted prospective clinical surveillance on one 20-bed general medical ward in our tertiary care academic medical centre (…).^32^ 3. “We aimed to assess a standardised handoff intervention’s effectiveness in reducing the AE frequency in paediatric intensive care units (PICUs) in a middle-income country using a stepped-wedge, cluster-randomised design.”^33^
Explanation Describing the study design (retrospective/prospective, observational/interventional) enables researchers and readers to understand the study’s strengths, limitations and validity of its findings. It also makes it easier to replicate the study or build on its findings. Researchers are encouraged to cite and adhere to additional EQUATOR Network reporting guidelines^1^ that are appropriate for their study’s study design such as STROBE^34^ for observational research or PRISMA for systematic reviews.^35^ SESAME is not intended to supplant those methodological reporting guidelines, but to increase the transparency and reproducibility in reporting on important elements in research relating to AEs and medical error. In the first two examples, the observational character is clearly addressed, as is the timing (retrospective/prospective). In the third example, an intervention is evaluated by using stepped-wedge, cluster-randomised design.

**Table:** 

6b Methods: study design—provide details of any patient or public involvement during any phase of the study (from design to dissemination) or state no involvement.
Examples 1. “Patients (…), a patient adviser (MD) and a caregiver adviser (AL) were involved in the design of the study, the design of the intervention, submission of the application for funding, monitoring of study conduct, interpretation of the data, review of the manuscript for important intellectual content and approval of the final manuscript for publication (…). When designing the intervention, input was sought from the patient and caregiver advisers, the emergency department patient advisory council and patients… regarding the clarity, helpfulness, and usefulness of the information included in the decision aid, and the decision aid was iteratively refined based on this input. (…).”^36^ 2. “Six patients who were members of our research advisory panel were involved in the development of our research questions, research protocol and selection of outcomes and they advised on the interpretation and dissemination of results.”^37^
Explanation Reporting patient or public involvement in a study provides transparency regarding how they have contributed to the research process at any stage of the study. This ensures that the research incorporates input from and shows respect for, the perspectives and experiences of those directly affected by the issue being studied, thereby enhancing the credibility of the research. Involving patients or the public helps ensure that the study addresses questions that are most relevant to those who are most impacted.^38,39^ Example 1 provides comprehensive detail about how patients and caregivers contributed across multiple phases of the study. Example 2 provides a succinct explanation of who was involved and at what specific stage and in a specific capacity. These both demonstrate transparent attribution of the roles and stages of involvement of patients, caregivers and representatives, avoiding vague statements simply noting that patients or others were consulted, which falls short of adequate reporting.

**Table:** 

7a Methods: setting details—describe the country/countries in which the study takes place or that is the source for data used.
Example “The study was set in Sweden, where assistance with activities in daily life is provided in patient homes by unlicensed staff (eg, assistant nurses) on behalf of the municipal social care services. The municipalities are also usually responsible for providing home healthcare to the elderly. Their healthcare organisations include unlicensed assistant nurses, physiotherapists and occupational therapists, with registered nurses (RNs) providing the highest medical competence (…). All physicians are employed by the county councils.”^31^
Explanation Reporting the country where the data were collected provides context to the study. Ideally, as in the example provided, this includes a concise explanation of information on the included country/countries that is relevant to the study, such as how the health service is structured and organised and availability of resources as these inform generalisability of study findings to other countries.

**Table:** 

7b Methods: setting details—describe the setting (eg, academic, community, both, urban, rural).
Example **“**We selected three large US tertiary care centers that had well-established operational patient safety programs based on the following criteria: (1) They received external funding for patient safety research; (2) they had internal operational programs focused on improved detection of safety incidents and adverse events through patient safety reporting programs, and special tools built around advanced electronic health record systems used within all three organizations; (3) and they had received external recognition for internal patient safety initiatives through awards, publications, or involvement in national initiatives. All three hospitals were part of large health systems. Hospital C was an academic hospital, and hospitals A and B were community-based teaching hospitals. Hospital A was located in a midsize metropolitan area in the West, hospital B was in a large metropolitan area in the Mid-west, and hospital C was in a large metropolitan area in the Northeast. Hospital size ranged from 550 to 1000 beds, and all three hospitals offered a full range of medical services, covering primary care to quaternary care. All three hospitals were major tertiary (specialized) referral centers with significant local market share and were teaching hospitals for their respective medical schools.”^40^
Explanation Reporting the setting of the study such as where the data was collected (eg, academic, community, both, urban, rural) provides valuable context. As is accomplished in the example above, this goes beyond naming the facility type to describe the organisational characteristics (teaching status, health system affiliation, safety programme maturity) that are relevant to interpreting the results. This assists in assessing the level of generalisability of the study findings. It also allows aggregation of data from multiple studies, where effects of setting on study findings can be explored narratively or statistically, for example, using meta-analytical methods and stratified analyses by the type of setting.^8,41^ When describing the setting, aim to include details that could influence event occurrence or detection—such as organisational complexity, staffing model, information systems or patient population. A description that states, for example, “the study took place at three hospitals” provides almost no context for interpreting results.

**Table:** 

7c Methods: setting details—describe the facility or unit type (eg, hospital, ambulatory, intensive care unit, nursing home) and volume (eg, single or multicentre, number of inpatient beds, annual visit volume, catchment area)
Example “Baylor Health Care System (BHCS) is an integrated health care delivery system in the Dallas–Fort Worth metroplex, serving patients in North Texas and beyond, and comprising 14 owned, leased, and affiliated hospitals and >190 primary, specialty, and senior health care ambulatory centers. AE data are presented here for the eight general acute care hospitals that have been part of BHCS since January 1, 2007, ranging in size and location from an urban 1,026-bed tertiary care hospital (~40 000 admissions per year), to a 69-bed community hospital (~4000 admissions per year) outside the urban metroplex.”^42^
Explanation Reporting the facility or unit type from where the data were collected provides valuable context. It helps illustrate geographic diversity and size, supporting assessments of representativeness and potential impacts on the interpretation of findings. The example above offers useful detail about the range of facility types, sizes, geographical locations and affiliation types, which enhances transparency. This information also allows aggregation of data from multiple studies, allowing the exploration of variability in study findings.^8,43^

**Table:** 

8a Methods: population details—describe any eligibility criteria related to a target medical condition or specialty (eg, oncology, orthopaedic, acute/elective care)
Examples 1. “Patients admitted (…) with an acute hip fracture over a 2-year period with intact cognitive function who were independent walkers pre-fracture, and willing and able to participate in the study, were eligible for inclusion.”^28^ 2. “Patients were eligible for the study if they were in the NICU (neonatal intensive care unit) for a minimum of 2 days and discharged, transferred out, or died in the NICU between November 1, 2004, and January 31, 2005.”^44^
Explanation Reporting the eligibility criteria related to a target medical condition or specialty of the study population clarifies the type of data collected. This should ideally include a rich description that aids in identification of the study group. This aids in the interpretation of fundings and assessments of generalisability. It also allows aggregation of data from multiple studies or comparison of results of studies to be undertaken at a more granular level or to be stratified. A systematic review of one type of AE data collection tool, the GTT, found that percentage of admissions with an AE in paediatric intensive care units ranged from 56–62% while in paediatrics the range was 9–34%.^7^ The difference in rates between the two specialties illustrates the importance of reporting medical condition eligibility criteria. Furthermore, some studies assess AE rates by hospital inpatients but may exclude certain specialties, such as psychiatry or rehabilitation.^45,46^ For purposes of generalisability and comparisons of data, these exclusions are important to report in these studies. Example 1 provides comprehensive details about a specific population and timeframe and their cognitive and functional status. Example 2 similarly specifies an explicit definition for the study population based on admission location, disposition and date range. Though listed in the heading as such, simply stating that the group was, for example, ‘oncology patients’ would fail to provide adequate information to aid in interpretation.

**Table:** 

8b Methods: population details—describe any eligibility criteria related to participant demographics (eg, adult, age, race, ethnicity, gender).
Examples 1. “This study included children aged <18 years who were admitted to hospitals between January 1, 2000 and December 31st, 2019 and underwent intermediate to high-risk surgery, according to previously described criteria. The child’s race and ethnicity were defined according to the HCUP (Healthcare Cost and Utilization Project) data element, based on categories defined by a 1977 Office of Management and Budget directive. We only included Non-Hispanic white, Non-Hispanic Black, and Hispanic children because limited mortality events in Asian, Pacific Islander, American Indian or Alaska Native patients precluded rigorous trend analysis in these groups.”^47^ 2. “The study was conducted by an integrative team of hospitalist physicians, nurse-reviewers, physician-adjudicators and researchers to determine the incidence and risk factors associated with post-discharge AEs. Eligible participants were recruited from the Tallahassee Memorial Hospital (TMH), an urban community hospital of Florida State University, from 14 December 2011, through 8 October 2012. Patients under the care of TMH physicians were recruited if they were being discharged home, spoke English and could be contacted 30 days after discharge for a telephone interview. Potential participants were identified from a specially run list of pending discharges, limited to those patients on the general medical service and under the care of a TMH hospitalist physician. In order to insure that equal proportions of rural and urban subjects were recruited, the study employed an approach successfully utilized by an earlier study of post-discharge adverse drug events that sought to ensure adequate enrollment of racial/ethnic minorities. Nurse-reviewers initially stratified the patient list by rural/urban using zip codes, then randomized the order in which potentially eligible patients were approached to ensure unbiased patient enrollment.”^48^
Explanation Like other setting and population details above, adequate reporting of participant demographic information for any targeted groups or conditions is important for interpreting study findings, particularly if demographic details comprised an important component of the study or comparisons between groups. This is also important for assessing generalisability and for comparing study findings across studies. Example 1 explains the rationale for what demographic data were included based on the ability to perform trend analysis, and in example 2, patient demographics were an important component in comparisons of postdischarge AE rates.

**Table:** 

9 Methods: exclusion criteria—describe any exclusion criteria for participants or events
Examples 1. “The records of patients who were under the age of 18 years and those who were admitted primarily for psychiatric or rehabilitation care were excluded.”^49^ 2. “We excluded AEs that were unrelated to the index admission and AEs that originated from the care of another AE. For example, if a patient was admitted because of a periprosthetic joint infection and sustained a fracture from falling in the ward, the infection was included as an AE, and the fracture was not included. We did not include planned outpatient visits at hospitals or planned or unplanned outpatient visits outside of hospitals, such as with a general practitioner.”^50^
Explanation Explicit descriptions of exclusion criteria clarify the target study sample. Like other setting and population details, reporting the exclusion criteria is important for assessing generalisability and comparison of study findings. Exclusion of specific events may alter the study estimates of AEs; for this reason, any such exclusion must be described. Example 1 provides a succinct description of ages and conditions for exclusion. Example 2 provides a more detailed description of the types of events that were out of scope and so were excluded.

**Table:** 

10a Methods: case finding/sampling strategy—provide details for the study period (eg, dates, recruitment period, duration).
Example “We used a stratified random sampling approach to invite general practices to participate from three different areas of England. We undertook a retrospective case note review of an open cohort of all primary care patients registered with participating general practices (between 1 April 2015 and 31 March 2016) to identify cases of avoidable significant harm (…).”^51^
Explanation Information on when a study was undertaken and how long it took can inform the context and generalisability of a study’s results and the methodology. Patient safety interventions and the environment in which studies take place change over time. Also, study methods may evolve over time. Issues such as seasonality could also impact findings. Understanding when a study took place and over what time period medical records were assessed or participants were recruited, together with other information on settings, places the study in historical context. How long the study took might be important methodologically informing issues such as challenges in patient recruitment or capture of AEs.^52^

**Table:** 

10b Methods: case finding/ sampling strategy – describe the method of case finding used (eg, all records meeting screening criteria; triggered records, computerised surveillance).
Examples 1. “Each month, the data analyst identified all records that met selection criteria and used a random-number generator to assign each record a number. The 20 inpatient records selected each month were based on the smallest assigned random numbers. From the randomized list of records, the first 20 meeting inclusion and exclusion criteria were chosen for review (…). Following the IHI protocol, the 2 trained reviewers read each record independently, limiting the review to 20 minutes regardless of length of stay or medical complexity. If none of the 53 GTT triggers were identified, the review process for that record was complete. However, if one or more triggers were noted, a more focused review of the record was conducted to determine if an AE had occurred based on the definition and criteria established by the IHI protocol.”^53^ 2. “We sampled patient records in three stages. In stage 1, we identified the total patient population of the practices at the start of the retrospective cohort (1 April 2015). In stage 2, we used electronic registry queries to identify patients at increased risk of significant health problems and/or avoidable significant harm (the ‘enhanced sample’) (…). In stage 3, one of the GP (general practitioner) data collectors screened the electronic health record of each patient in the enhanced sample to identify any new significant health problems experienced by patients over the 12 months of the study (1 April 2015–31 March 2016).”^51^
Explanation The process for case finding and sampling strategy is important for understanding the validity, reproducibility and generalisability of the study. Different methods of sampling, including whether triggers are assessed manually or electronic algorithms are used, can yield different results in terms of frequencies and types of AEs.^13,54,55^ Where appropriate, this description should also include sampling strategy (eg, consecutive, random, convenience). The description of case finding methods needs to be sufficiently detailed to allow for the replication of the study, an assessment of validity and to determine the extent to which the case finding / sampling strategy leads to a study cohort that represents the target population.

**Table:** 

11a Methods: reviewers and training—describe reviewer recruitment, qualifications and experience in event adjudication.
Example “The patient records were reviewed by a team of 66 nurses and 55 physicians. The team of recruited physicians consisted of 25 general internists, 20 general surgeons, 5 neurologists and 5 paediatricians. Most were recently retired. Recruitment of the physicians started through personal contacts of the project leader and was extended with contacts with the scientific association of internal medicine, surgery, neurology and paediatrics. The selection criteria for physicians were: at least ten years post graduate general clinical experience; good reputation among colleagues; no longer than five years retired; experience or affinity with analysis of incidents, complaints and errors; availability for at least one but preferably two days per week. The nurses were recruited via the website of the association for nurses and websites of hospitals. The selection criteria for the nurses were: minimum five years clinical experience; experience or affinity with analysis of incidents, complaints and errors; availability for at least one, but preferably two days per week.”^56^
Explanation It is important to understand what qualifications a reviewer has for performing reviews. Reviewer performance improves over time and experience matters. Clinical expertise in a content area alone without an understanding of, or experience in event determination is likely insufficient and will likely impact event detection.^57,58^ Conversely, reviewers who may no longer work clinically could potentially lose insight into important considerations that are part of current practice. Selection bias in reviewer recruitment type may also impact findings. The example above provides a description of attributes of review team members, their experience, clinical areas and how they were recruited. A description of experience in event adjudication that includes prior formal training or specific roles or responsibilities in this area would provide additional assurances. An explanation of why and how reviewers were recruited and any deliberate decisions in achieving the desired balance or attributes of the review team is therefore highly important. This allows the reader to conclude whether potential biases exist and might affect findings.

**Table:** 

11b Methods: reviewers and training—describe reviewer training and what performance standards for reviewers (if any) were required prior to independent review.
Examples 1. “The nurses and physicians followed a one day training in small groups (max 12 participants) led by one member of the research team and one experienced nurse or physician, respectively. During the training, the study protocol, definitions and review forms were explained and examples of (preventable) adverse events were discussed. The reviewers practiced with cases and the review forms and they were provided with a review manual. At the end of the training, the nurses underwent a reliability test. After one month of reviewing, the reviewers had a half-day training session to discuss their problems concerning the review process and definitions and to update the reviewers with the latest insights about the review process. These training sessions were frequently repeated during data collection. The discussed problems were collected in a regularly updated Frequently Asked Questions (FAQ) document which was regularly handed out to all reviewers.”^56^ 2. “The PACHMT (Pediatric All-Cause Harm Measurement Tool) trigger list (table 1), an instruction manual containing detailed definitions of triggers, a list of likely associated predefined harm events and case examples, and data collection forms were distributed to each hospital. (…). All clinical reviewers and physician reviewers from each site completed standard training on trigger tool chart review methods incorporating the PACHMT and based on previous work by the Children’s Hospital Association and IHI. Training was taught by IHI GTT expert educators and consisted of 3 interactive educational webinars. During these sessions, these experts described trigger detection, introduced the PACHMT trigger list, presented the process for the standard application of the tool, reviewed examples and facilitated active dialogue. The site reviewers independently completed standard case studies for detecting triggers, harm identification and harm classification that were reviewed during training sessions. Calibration exercises, such as extensive case reviews and group review of event and harm classifications, during training assisted in promoting consistency in the use of the tool and study definitions.^59^ 3. “κ scores will be calculated to test the level of agreement between each surveyor and one of the CTK (CareTrack Kids) researchers. Each surveyor must achieve a κ score of 0.8 before collecting data. After the first 2 weeks of data collection, another IRR (inter-rater reliability) test will be undertaken to assess progress. IRR results of 0.8 are acceptable for the surveyor to continue. Surveyors scoring less than 0.8 will be provided with training and re-evaluated. Surveyors unable to achieve this target will be redeployed within the project.”^60^ 4. “As described in our prior studies, the review process consists of rigorously trained reviewers (ED Nurses, ED Acute Care Nurse Practitioners, ED Physician Assistants and Emergency Physicians), whose training included review of prior EDTT papers, the Institute for Healthcare Improvement Global Trigger Tool white paper and training modules, modified Global Assessment of Pediatric Patient Safety Trigger Tool training modules, and review of a set of standardized cases we previously developed. Reviewers had to achieve 80% sensitivity in detecting adverse events in test batches for qualification. Second-level physician reviewers were all board certified in emergency medicine, with more than 15 years’ experience working in quality and safety roles.”^61^
Explanation Multidisciplinary teams can add helpful perspectives on event adjudication, but it is important that review teams have a shared mental model to work from in assessing concepts such as harm and preventability.^62–64^ Even seasoned clinicians may at times conflate constructs such as ‘error’ with ‘harm’ or may not be adequately tempered in deliberations where the insidious nature of hindsight and outcome biases impact judgement. Specific training in event detection is critical for optimising reviewer performance and to improve within-reviewer and between-reviewer reliability. Researchers should describe training in sufficient detail, together with any assessment of the success of training. This may include the measurement of reviewer performance compared with some reference standard.^58,65^ Different measures of agreement exist (eg, Cohen’s kappa and intraclass correlation coefficients). Example 1 provides a thorough description of the content of training. Example 2 describes the content and format of training and exercises to calibrate performance. Examples 3 and 4 define specific thresholds that are required as part of reviewer credentialing. Detailing the rigour of training is essential to convey to readers that results are accurate, reliable and thus credible.

**Table:** 

12a Methods: review process—describe how reviews were conducted (eg, by an individual, multiple reviewers working independently or in phases or together as a group) and any consensus technique used.
Examples 1. “(…) the 2 trained reviewers read each record independently (…). If none of the 53 GTT triggers were identified, the review process for that record was complete. However, if one or more triggers were noted, a more focused review of the record was conducted to determine if an AE had occurred based on the definition and criteria established by the IHI protocol (…). After their independent review of the 20 records each month, the nurse reviewers met to compare their results and reach consensus on the identification of triggers, AEs, and harm. They then met with the physician member of the review team, who made the final decision of whether an AE occurred and the severity of harm associated with that event.”^53^ 2. “Data collection involved a three-stage medical record review. In the first stage, the nurses assessed each record for the presence of at least one of 18 criteria, indicating a potential adverse event (table 1). To assess inter-rater reliability, 10% of the medical records were screened by two nurses. In Stage 2, each record that was positive for one or more criteria was reviewed, independently, by two physicians. (…). After having performed independent assessments and completing the case report forms, the physicians discussed their findings with the aim of verifying complete and correct retrieval and interpretation of the information in the medical records. The focus of the discussion was on the assessment of causation and preventability. The physicians were instructed to indicate clearly any change in their original judgment, subsequent to their discussion, in the case report forms. When the physicians had different opinions concerning the presence of an adverse event or its preventability, an independent assessment was performed by a member of the Scientific Council of the National Board of Health and Welfare (Stage 3).”^66^
Explanation The review method used in a study is highly impactful on findings and details around this important aspect of methods are critical. Many studies use a two-stage review process, sometimes with dual independent reviewers in stage 1 and a secondary confirmatory stage.^8^ This is the approach recommended by the GTT. Yet, in a systematic review, only 27% of GTT studies adhered to this approach, with 17% using a single first level reviewer and 23% not specifying their approach at all.^7^ Among studies not using the GTT, some use single reviewers, others use single or dual independent review with arbitration by a third reviewer or a group in the case of disagreements and still others include group reviews.^55,67,68^ Review by more than one person might capture more details and offer a broader set of perspectives, with high levels of agreement demonstrated in some studies but without any direct comparisons we are aware of presenting differences in sensitivity.^62–65,69^ The examples above provided describe their processes in detail, aiding the reader in understanding the process by which determinations were made. This may include evaluation of an event by a single individual or by multiple individuals reviewing the same event. The latter may include a concurrent group review or, commonly, a tiered review, with one or more reviewers looking for events in a record that are then confirmed by a second level reviewer, which is the process recommended for use with the GTT.^5,6^ Single review obviates the need for evaluation of interrater reliability but is prone to individual biases. Group reviews may conversely be prone to dominance bias or conformity bias/groupthink. It is known that sensitivity is improved with multiple reviewers, so that the number of missed events is reduced.^57,58,62^ This method would be preferred to enhance capture but must be weighed against potential resource constraints and the specific emphases of a studies (eg, sensitivity vs specificity). Safeguards such as audits, periodic dual review and calibration checks can also be used to mitigate bias. Because the details of the review method used are essential for rigour and transparency, reporting on these is critical. In the examples, the review processes are well explained, which also facilitates replication.

**Table:** 

12b Methods: review process—describe any time limit on the duration of reviews, as this may impact outcome estimates (eg, a maximum of 20 min per review).
Examples 1. “Following the IHI protocol, the 2 trained reviewers read each record independently, limiting the review to 20 minutes regardless of length of stay or medical complexity.”^53^ 2. “The electronic patient records were reviewed in two stages (…). In Stage 1, the neonatal registered nurse reviewed all professionals’ notes, without any time restrictions, based on 88 predefined triggers, for example, abnormal body temperature or tissue harm.”^41^
Explanation Spending more time reviewing an individual record often translates to finding more events. Limitations on the review time are frequently applied to enhance the feasibility of the study and to normalise the review process for better reliability and generalisability. For example, the IHI’s GTT recommends truncating reviews at 20 min.^6^ However, time is but one parameter in understanding review duration. The nature of materials being reviewed in some period of time will significantly impact event detection and reproducibility. Navigation through relevant sections of a record—whether on paper forms or in electronic records—can be impacted by how information is organised. In the time it takes to review a specific section of a weeks-long inpatient visit, a reviewer might be able to examine the entirety of one or more complete emergency department or outpatient visit records. Therefore, a detailed explanation of the scope and nature of a review helps readers understand what was undertaken to help shape their sense for comparability and reproducibility across studies.

**Table:** 

12c Methods: review process—describe how individual events were determined (eg, implicit or explicit review, use of an algorithm, use of automated/machine learning approaches).
Examples 1. “Structured implicit review assisted physician reviewers to assess causation and preventability with the tool guiding reviewers through a series of questions before they made their judgements.”^70^ 2. “The automated adverse event detection process involves several sequential steps. First, automated trigger reports are generated from various hospital information systems on a nightly basis. Second, each trigger report is examined by a clinical analyst to determine if an adverse event occurred, the level of harm associated with the event, and whether it was preventable. This investigation occurs via review of the EHR (electronic health record), any associated paper records, and via interviews with providers caring for the patient as appropriate. Third, adverse event data detected through automated detection are entered into an institution-specific database if not already entered into the voluntary incident reporting system. Finally, aggregated data are analyzed and presented on a monthly basis to an institution-specific Automated Adverse Event Detection Steering Committee.”^71^
Explanation Apart from the mechanics of the review process, the details of how an individual review is conducted are crucial for understanding a study and its findings. *Implicit review* is essentially a reviewer’s subjective judgement, comparing circumstances being reviewed with one’s own knowledge, opinions and beliefs. In *structured implicit reviews*, reviewers apply their internalised standards but are directed to base their judgement on specific issues and information. *An explicit review* uses detailed specific criteria or rules that help define the elements and framework used in making determinations, for example, as to whether an event occurred. Some approaches use an algorithm to determine whether an event is considered, for example, an adverse drug event.^55,72,73^ Computerised surveillance can increase yields and is based on prespecified criteria. However, the explanation of the foundations and considerations, inputs and logic on which algorithms are constructed is important as these can impact results.^14,74,75^ Ultimately, beyond assigning a name to the review process used, investigators should aim to describe: (a) how determinations were made, (b) what standards or criteria guided those judgments and (c) how consistency or reliability was ensured.

**Table:** 

13a Methods: event scope and definition—indicate the outcome(s) of interest (eg, all-cause harm, preventable or ameliorable harm, errors, near misses, non-harm events) and include explicit definitions for the outcome(s) of interest. If harm is the outcome, indicate whether the definition of harm requires that an intervention take place.
Examples 1. “Primary outcomes were the occurrence of AEs and AE rate (AE/PICU day).”^76^ 2. “All no-harm incidents that were detected in the home healthcare records during the review were included, regardless of if they originated from home healthcare or other healthcare or social care organizations.”^77^ 3. “Consistent with recent work, we defined AEs as unintended physical injury (resulting from or contributed to) by medical care that required additional monitoring, treatment, or hospitalization, or that resulted in death.”^78^ 4. “In accordance with definitions used previously, an adverse event was defined as (1) an unintended injury or complication, which results in (2) disability at discharge, death or prolongation of hospital stay and (3) is caused by healthcare management (including omissions) rather than the patient’s disease.”^66^ 5. “An AE was defined as suffering, physical harm or disease as well as death related to the index admission and as a condition that was not an inevitable consequence of the patient’s disease or treatment.”^50^ 6. “(…) a no-harm incident was an event caused by healthcare or social care that reached the patient and could have led to an AE but resulted in no discernible harm.”^77^
Explanation It is important to indicate the scope and definition of event types that are being evaluated. This may be restricted to what is assessed to be preventable harm or may include all-cause harm, regardless of preventability. Whether a study’s focus includes near misses or non-harm events is important so that readers understand what outcomes are in scope and what impacts this has for any comparison purposes. The first two examples illustrate the scope/outcome of interest. While the Standard Elements in Studies of Adverse Event or Medical Error checklist does not prescribe a single definition, it is important to understand and convey to readers that varying definitions of an outcome such as ‘adverse event’ can make significant differences in study findings. For example, some definitions of an AE, such as that used by the GTT (which does not consider acts of omission as AEs) require that some intervention(‘ (…) additional monitoring, treatment or hospitalisation’) is undertaken that addresses the harm in order to be considered an AE as can be seen in example 3. An allergic reaction or borderline blood glucose or borderline hypoxia that is monitored rather than intervened on (with an antihistamine, dextrose or oxygen administration respectively) is not considered harms in this framework. Approaches that do include acts of omission, such as that used in the HMPS method, do not require an intervention. In this case, failure to administer antibiotics in sepsis or failure to give prophylaxis in a hospitalised patient who develops a thrombus would likely be considered AEs. On the other hand, the HMPS method requires that an AE results in disability, death or prolonged hospital stay, as illustrated in example 2. This criterion in the AE definition excludes minor but common AEs.

**Table:** 

13b Methods: event scope and definition—describe the causation standard used when determining events (eg, ‘caused by’ vs ‘resulting from or contributed to by’ healthcare) and specify whether a scale or rating system was used to characterise the confidence of causation and if so, describe this and its origin.
Examples (derived from the HMPS methodology) 1. “Causation was present if the AE was caused by health care management rather than the disease process. A scale from 1 to 6 was used to determine whether an AE was caused by health care management or the disease process. 1=Virtually no evidence for management causation 2=Slight-to-modest evidence for management causation 3=Management causation not likely, less than 50–50 but close call 4=Management causation more likely than not, more than 50–50 but close call 5=Moderate/strong evidence for management causation; and 6=Virtually certain evidence for management causation.”^79^ 2. “To qualify as an AE/no-harm incident, a score of 3 or higher on a 4-point Likert Scale was required (1=the AE/no-harm incident was not related to healthcare/social care, 2=the AE/no-harm incident was probably not related to healthcare/social care, 3=the AE/no-harm incident was probably related to healthcare/social care and 4=the AE/no-harm incident was related to healthcare/social care).”^77^ 3. “The objective of the second stage was to determine whether the sampled admission was associated with an adverse event (…). Review Form 2 (RF2) guided these judgements (…). Both review forms were closely modelled on the comparable instruments in the American and Australian studies. The degree of certainty accorded to this assessment was translated into a six-point confidence scale of evidence of causation by healthcare management. This ranged from 1=virtually no evidence, to 6=virtually certain evidence.”^80^
Explanation While no approach to AE evaluation considers morbidity or mortality due to a patient’s underlying condition to be an AE, the standard used for determining whether an event is healthcare-associated is of critical importance. Whether researchers use the standard that an event is ‘caused by’ healthcare or ‘resulting from or contributed to’ by healthcare can make a significant difference in harm estimates.^11,12^ Some approaches, such as the HMPS methodology, use scales to determine confidence in such decisions as shown in the examples. Researchers should describe the causation standard applied and indicate whether a scale or rating system was used. If so, they should specify which scale was used, reference its original source, and note any modifications made.

**Table:** 

13c Methods: event scope and definition—provide the time frame(s) for event inclusion (eg, present on arrival, during the index visit, some period of time after an intervention).
Examples 1. “To be included as an AE or no-harm incident in the study, one of the following criteria had to be met: 1. The AE or no-harm incident occurred during the index admission, that is, within 90 days after enrolment in home healthcare, regardless of caregiver. 2. The AE or no-harm incident derived from caregivers outside home healthcare (outpatient care, social care or in-hospital care) occurred within 30 days prior to the index admission and was detected during the index admission.”^77^ 2. “To be included as an AE in the study, one of the following three criteria had to be met: (1) The AE occurred within 30 days before index admission, caused the index admission, or was detected during the index admission; (2) the AE occurred and was detected during index admission; (3) the AE occurred during index admission and was detected within 30 days of index discharge from the children’s hospital. Adverse events identified by using the latter criterion were not required to result in a new admission; in other words, an AE treated on an outpatient basis was included.”^81^ 3. “The AE had to occur within a 3-month period before or during the index admission and be detected during or within the 3-month period before or after the admission.”^82^
Explanation Many approaches to event detection limit the time frame during which an event occurs, in order that the event may be plausibly attributable to some initial action. For example, a fall that is purportedly related to administration of an opioid should probably occur within a time frame (hours) when the medication is still active in order for the fall to be reasonably attributable to the medication. Similarly, a return to the emergency department for some event should occur within a specified time frame in which the event can be reasonably attributed to some prior action or inaction in order to be considered an AE. Specifying this is important for establishing the scope of the study. The time frame(s) used may impact findings and is important for comparisons across studies.^13^

**Table:** 

13d Methods: event scope and definition—indicate whether event capture included acts of omission, acts of commission or both.
Examples 1. “AEs related to acts of either omission or commission were included.”^50^ 2. “IHI further defines events causing harm as including only those AEs related to the active delivery of harm (commission) and excludes issues related to substandard care (omission).”^53^
Explanation Some approaches to event detection, such as the IHI GTT, specifically exclude acts of omission.^6^ This is due to the possibility that the requirements could shift over time, as advances in medicine and technology may alter what actions are considered necessary each year. Others feel that acts of omission are particularly susceptible to outcome and hindsight biases and that their inclusion may lead to inappropriate and inaccurate findings. However, other approaches do include acts of omission as AEs, feeling they are an important source of learning for improvement.^83^ For example, if clinicians made the correct diagnosis but failed to provide treatment and where there is a consensus that this reflects omission of a standard of care (eg, failure to give antibiotics after diagnosing sepsis), that this is considered an AE and an act of omission. Researchers should indicate whether they include acts of omission as AEs in their methods, as done in the examples, as this may impact the results and is important for comparisons across studies.

**Table:** 

13e Methods: event scope and definition—indicate whether ‘cascading’ events, where one event caused the next, were counted as a single event or each event was counted separately.
Example “Initial events progressed and triggered other related harm events that resulted in the patient’s death. For example, one patient acquired a methicillin-susceptible Staphylococcus aureus (MSSA) infection during the hospital stay. The infection led to septic shock, which contributed to the patient’s death. Another cascade event involved substandard treatment of sepsis with insufficient fluid administration and inadequate antibiotic treatment, which led to septic shock, respiratory failure and the patient’s death.”^84^
Explanation Cascading events are where one event causes the next or where they have the same root cause. This idea prevents inflation of event numbers by identifying these as a single event. Indicating how such events are considered and counted in analyses is therefore an important item impacting study findings and so describing this is essential for transparency and comparisons across studies.

**Table:** 

14a Methods: characterisation of events—describe the taxonomy or classification system used to describe the event and/or contributing factors (eg, surgical/procedural events, health care-acquired infections, adverse drug events).
Examples 1. “We will use the comprehensive PatIent SAfety (PISA) classification system to deconstruct the case narratives written by the GP reviewer(s) to record the type of patient safety incident, contributory incidents, contributory factors and severity of harm. We will also identify the chronological sequence of events leading up to an incident by drawing on the recursive model for incident analysis, to allow us to build a structured and coded sequence, using codes from several classification frameworks.”^85^ 2. “The WHO International Classification for Patient Safety (ICPS) (…) contains ten high-level conceptual areas, of which six areas (i.e. patient characteristics, incident type, incident characteristics, contributory factors, actions to reduce risk, and ameliorating actions) were adapted for this study. Three of the remaining four areas of the ICPS (i.e. detection, organisational outcomes and patient outcomes) were either not able to be identified or not relevant for this study.”^86^
Explanation At least in theory, capturing and categorising events helps to identify ‘hot spots’ in need of quality improvement. Many taxonomies focus on the errors that lead to AEs, while others centre on the harm experienced by the patient, and some adopt a more hybrid approach. Since there is no single agreed-upon taxonomy to describe event types and/or contributing factors, it is important to clearly describe the approach taken in a study, including any adaptations made to suit the specific context. Any adaptation of an existing taxonomy should be described clearly, including changes in category definitions. In the examples, the classification systems are named, and an adaptation is described in the second example, where some areas are omitted.

**Table:** 

14b Methods: characterisation of events—describe whether a severity assessment was made and if so, indicate what scale was used and its origin.
Examples 1. “The severity of the AEs was evaluated using a slightly modified version of the National Coordinating Council for Medication Error Reporting and Prevention (NCC MERP) index. NCC MERP index categories E–I were included, and the categories indicated the following: (E) contributed to or resulted in temporary harm, (F) contributed to or resulted in temporary harm that required outpatient or inpatient care or prolonged hospitalisation, (G) contributed to or resulted in permanent harm, (H) required intervention necessary to sustain life within 60 min and (I) contributed or resulted in the patient’s death.”^50^ 2. “The second severity scale was that used in the Harvard Medical Practice Study (HMPS) and subsequently in several nationwide AE studies. It encompasses seven grades (minimal impairment, recovery within 1 month; moderate impairment, recovery within 1–6 months; moderate impairment, recovery within 6–12 months; permanent impairment, degree of disability≤50%; permanent impairment, degree of disability>50%; contributed to patient death; unable to determine).”^31^
Explanation Most studies of AEs assess severity to show their effect on patient outcomes and prioritise safety improvements. At times, this is called ‘disability’ or injury. It typically uses ordinal scales ranging from no harm to permanent disability or death. There are also gradations of non-harm events. The National Coordinating Council’s Medication Error Reporting and Prevention Index^87^ is one such scale used especially for medication-associated harm, but there are others in use such as the HMPS scale which grades impairment based on recovery time, making it suitable for retrospective reviews.^5^ Researchers should indicate whether a severity assessment was made and if so, what scale they used in their study, cite to its origin and note any modifications. Ideally, this should also include an explanation of what specific construct or dimension (eg, functional impairment, resource utilisation, etc) this represents. The examples show that the two studies used different classification systems, which are to be taken into account when interpreting the study findings.

**Table:** 

15a Methods: preventability—indicate whether preventability was assessed and if so, provide the explicit definition used.
Examples 1. “(…) preventable AE was defined as an event that could have been prevented if adequate actions had been taken during the patient’s contact with healthcare.”^50^ 2. “Preventable events were defined as “events where definite breach of standard professional behavior or technique was identified; necessary precautions were not taken; event was preventable by modification of behavior, technique or care.”^59^ 3. “A preventable AE results from an error in management due to failure to follow accepted practice at an individual or system level. Accepted practice was taken to be “the current level of expected performance for the average practitioner or system that manages the condition in question.”^76^ 4. “Most studies classify patient harm as preventable if it occurs as a result of an identifiable modifiable cause, and its future recurrence can be avoided by reasonable adaptation to a process, or adherence to guidelines.”^8^ 5. “Answering yes to one or more of the following questions suggests that the adverse drug reaction (ADR) in question may indeed have been preventable: 1. Was the drug involved in the ADR not considered appropriate for the patient’s clinical condition? 2. Was the dose, route and frequency of administration not appropriate for the patient’s age, weight and disease state? 3. Was required therapeutic drug monitoring or other necessary laboratory Test not performed? 4. Was there a history of allergy or previous reactions to the drug? 5. Was a drug interaction involved in the reaction? 6. Was a toxic serum drug level documented? 7. Was poor compliance involved in the reaction?”^88^
Explanation Assessing preventability may help prioritise safety improvements by identifying harms that could be avoided through better systems or practices. It supports targeted, patient-centred interventions and promotes system learning. However, preventability is context-specific and may change over time as technologies and care processes evolve. Further, the details of what is meant by preventability can have significant impacts on findings.^10,89^ Some tools, like the GTT, avoid such assessments altogether, stating that today’s unpreventable event may be an innovation from being preventable in the future.^6^ If used, researchers should clearly define what they mean by ‘preventable’. This should ideally include whether an event is preventable in theory, under ideal conditions or in the actual context since this affects the interpretation and validity of study findings. The examples illustrate several definitions of preventability in diverse clinical contexts.

**Table:** 

15b Methods: preventability—describe any preventability scale or rating system used, its origin and the language used for various ratings (eg, definitely not preventable, possibly preventable vs potentially preventable).
Examples 1. “Preventability was assessed using a similar 4-point Likert scale as follows: (1) the AE was not preventable, (2) the AE was probably not preventable, (3) the AE was probably preventable and (4) the AE was preventable. AEs that were graded 3 or 4 were classified as preventable.”^50^ a. “The degree of preventability of AEs was measured on a six-point scale, grouped into three categories: No preventability 1.(Virtually) no evidence for preventability Low preventability 2. Slight to modest evidence of preventability 3. Preventability not quite likely (less than 50/50, but ‘close call’) High preventability 4. Preventability more than likely (more than 50/50, but ‘close call’) 5. Strong evidence of preventability 6. (Virtually) certain evidence of preventability”^56^ 3. “In order to allow for comparisons between studies, Incidence and preventability were measured through the application of a protocol developed by the researchers responsible for the Ibero-American Study of Adverse Events (IBEAS), which had as a precursor the protocol developed by the HMPS. In addition, we used the method of retrospective review of medical records proposed by researchers from the Canadian Adverse Event Study (CAES), one of the most relevant derivations of the HMPS method.”^90^
Explanation Reviews have identified a wide range of preventability rating systems in the literature, which vary in the extent to which they incorporate clinical judgement and structured criteria.^7,55,67^ Common examples include the 6-point Likert-style from the HMPS scale, the Hallas criteria^91^ and the WHO P-Method. Researchers should report the exact wording of preventability categories, as these factors influence interpretation, analysis and the reliability of findings. If a published scale is used without any adaptations, researchers should cite the originating article, and/or name the scale.

**Table:** 

15c Methods: preventability—describe the reviewer process used in determining preventability (eg, single or tiered review, single or dual reviewers per tier, group review).
Example “In Stage 2, each record that was positive for one or more criteria was reviewed, independently, by two physicians. (…). Similarly, preventability of an adverse event was assessed using a scale from 1 to 6. At ratings of at least 4 (ie, more than 50% likelihood), adverse events were classified as preventable. (…). After having performed independent assessments and completing the case report forms, the physicians discussed their findings (…). The focus of the discussion was on the assessment of causation and preventability. (…). When the physicians had different opinions concerning the presence of an adverse event or its preventability, an independent assessment was performed by a member of the Scientific Council of the National Board of Health and Welfare (Stage 3).”^66^
Explanation If a preventability assessment is conducted, researchers should specify whether it was determined by a single reviewer or multiple reviewers, if assessments were conducted independently or jointly, and whether a tiered (phased) or consensus process was employed. Use of multiple reviewers and structured arbitration is expected to enhance validity. Clear reporting of the review process is essential to evaluate the credibility of preventability judgments. The example explains well how many reviewers were involved, that the reviewing was independent, and how consensus was reached.

**Table:** 

16a Methods: quality assurance—indicate any deviations from the intended study plan.
Example “Move to a Single Record Review. In October 2010, AHS (Adventist Health System) tested a single record review process after 17 months’ experience with double-record review. To ensure that the single-record review did not affect harms detection, we compared the percentage of harms of F or greater (F+) detected for six months before and six months after introducing the new process—that is, 40% vs 49%, which led us to adopt the single-record review process.”^92^
Explanation In the example above, recognising that methodological changes can influence study findings, the researchers not only amended their study method but also assessed the impact on harm detection rates before finalising the change. Deviations from a study plan may be necessary, for example, to improve feasibility or statistical precision when event prevalence is lower than expected. These can include changes to inclusion criteria, sample size or outcome definitions. Since such deviations can affect the validity and generalisability of results, they should be clearly reported. Transparency allows readers to understand what was actually done, supports replication and improves interpretation. Fully disclosing deviations—whether planned or not—is essential to ethical, rigorous research and helps identify strengths or weaknesses in study design or implementation. Failing to report them may lead to misrepresentation or overinterpretation of results. Even seemingly minor deviations are essential to report, as they may contribute to differences in outcomes across studies.

**Table:** 

16b Methods: quality assurance—describe any quality assurance mechanisms to improve validity and reliability (eg, monitoring/auditing, feedback provided to reviewers).
Examples 1. “All review teams were supported by record review experts in the research group who could answer questions. To ensure review quality, one expert monitored all reviews from the primary and secondary review stages for completeness and adherence to the trigger and adverse event definitions, and project manual. Any questions or discrepancies were referred back to the relevant team for resolution to make sure that the adverse event inclusion followed the project manual.”^31^ 2. “Routine quality checks were carried out to improve the quality of information gathered. During both the screening and review stages, forms were checked for completeness and adequacy. During the review stage, (nurse and physician) discrepancies in judgement of criteria presence or AE determination were checked and, if necessary, forwarded for adjudication to the Expert Reviewer. At the stage of data entry from the completed forms, standard checks for invalid, out-of-range, inconsistent and missing data were used to identify errors. Where medical knowledge was necessary, the Expert Reviewer was consulted.”^93^
Explanation Record review studies rely on the accurate extraction and interpretation of data from patient records. Quality assurance ensures that data is collected correctly, reducing the risk of errors or misinterpretations that could affect the study findings and conclusions. Quality assurance intends to ensure that the review process is consistent across and within different reviewers and records. This should increase the reliability of the results, making sure that similar events are assessed in the same way and reduce variability that could compromise the study’s validity. Quality assurance can be implemented through various activities, including training sessions and study manuals (standard operating procedures), expert support for reviewers in both methodology and context, monitoring and auditing, written and/or oral feedback and reviewer meetings. However, quality assurance should be proportionate to the scale of the study, what is sufficient for a local review may be inadequate for multisite designs. The examples provided illustrate some of the existing quality assurance mechanisms. In example 1, quality assurance is explained by review expert oversight in the review process and the access to review experts when needed. Example 2 outlines a similar quality assurance approach, offering some specific examples and emphasising the expert reviewer’s involvement in both clinical consultation and adjudication.

**Table:** 

16c Methods: quality assurance—include assessment(s) of inter-rater reliability for outcomes in the study (eg, whether an event represented harm, its preventability and categorizations) and describe how assessments were conducted and calculated.
Example “Inter-rater reliability was evaluated in review stage 1. Six percent of the records (n=125 records including 151 potential AEs) were randomly selected and double-reviewed to assess agreement between the primary reviewers’ judgments concerning whether at least one trigger or potential AE was identified in the record, if the record was to be forwarded to secondary review, if they found the same specific event and if this event was a potential AE. The reviewer pair comprised of one skilled and one less skilled in using structured record review. The judgments in the double-reviewed records were discussed between reviewers to reach consensus and to form the basis for reviews in stage 2. (…). No double review was performed in review stage 2 since a thorough monitoring process was used. (…). Cohen’s Kappa (Cohen, 1960) was calculated for inter-rater reliability between the primary reviewers.”^94^
Explanation In medical record reviews, inter-rater reliability is a quality control measure that assesses how consistently reviewers interpret the same case using defined variables. High inter-rater reliability indicates consistent application of criteria and strengthens the validity and credibility of findings. Low reliability suggests variation in interpretation, possibly due to unclear definitions (eg, of “adverse event”) or inadequate training. It highlights the need for clearer manuals, better training or revised tools. Monitoring inter-rater reliability during both training and data collection helps ensure consistency. However, even high agreement can be misleading if reviewers deviate from the protocol, underscoring the need for monitoring throughout the review process. Cohen’s Kappa (featured in the example) represents only one among several methods for estimating inter-rater reliability. Authors should clearly identify the method(s) used and, as illustrated in the example, describe the precise conditions under which testing was carried out. Beyond numerical reporting, contextual information (eg, frequency of checks, retraining) adds interpretive value.

**Table:** 

16d Methods: quality assurance—for any automated electronic data capture, describe whether there was any process to validate data against manual review.
Example “The web-based abstraction application displays patient demographics and a complete list of triggers (figure 1). Reviewers indicate whether each trigger was present (yes/no) on their manual review of a record (eg, the query says heart rate (HR)>130. The manual reviewer confirms whether there was a HR documented as>130 in the record or not). After this manual screening is completed, the interface displays the query results side by side with the completed manual review results and (reviewers) are then able to recheck the record and modify their responses if desired. (…). During this query validation phase of the study, (second level) reviewers also completed manual screening for triggers with both (first level) reviewers’ and computer query results visible, and arbitrated disagreements with the computerized query and/or with one another.”^95^
Explanation Use of various automated data capture methods (queries, algorithms, artificial intelligence) is increasingly used to replace manual approaches. In the example, the method applied was to compare automated extraction to manual review to assess trigger capture rates. In many AE studies, automated or trigger-based approaches are benchmarked against manual review by trained clinicians, which serves as the reference standard. Researchers should describe any measures applied to validate the findings of electronic data capture. The description of the comparator sample, reporting of concordance rates, and how discrepancies were handled strengthen the guidance and enhance interpretability.

**Table:** 

17a Methods: data analysis—describe all statistical methods used, including whether this study includes a post-hoc analysis and how missing data were handled.
Examples 1. “To compare AE rates per 1000 patient days and 100 admissions among hospital types (teaching vs nonteaching) and patient groups with chronic condition indicators (CCIs), we performed Poisson regression models. Additionally, we adjusted for CCIs to compare between teaching and nonteaching hospitals because teaching hospitals had a significantly higher proportion of patients with≥1 chronic condition than nonteaching hospitals. We had complete data on chronic conditions from 15 of 16 hospitals. To assess changes in the number of AEs per 1000 patient days and AEs per 100 admissions over time, we performed Poisson regression models with hospital random intercepts to account for hospital-level clustering and a term indicating hospital discharge date (24 quarters over 6 years). To account for any temporal changes in AE rate due to changes in patient demographics and chronic illness over time, we conducted additional regression analyses, controlling for age, sex, insurance, and CCIs.”^78^ 2. ”This study was a post hoc analysis of the TEAM trial (…). All patients, with available data, from the TEAM trial were included. Continuous data were reported as median (quartile 25th–quartile 75th) and compared using the Wilcoxon rank-sum test or Kruskal–Wallis test. Categorical data were reported as numbers (percentages) and compared using the Fisher’s exact test. The relationship between adverse events and patient survival was analysed using a Cox regression frailty model to account for clustering at a site level, with adverse events treated as a time-dependent variable and presented as a Kaplan–Meier survival curve with a corresponding subdistributional hazard ratio (95% confidence interval (CI)). A p value<0.05 was considered significant. No adjustment for the multiplicity of testing was performed. (…).”^96^
Explanation Key principles guiding the level of detail in reporting on analyses include transparency, avoidance of bias and enabling verification or reproduction of results, which applies to Item 17a but also to Items 17b–17e. Whereas descriptions are best kept short and concise in the methods section, sufficient detail is to be provided in an appendix or online repository to ensure reproducibility, allowing an independent statistician to reconstruct core analyses. While detailed guidance on reporting depth cannot be precisely defined, researchers should clearly state the metric or estimand used for each outcome and explain how estimates were derived from the statistical models in the methods sections. All outcomes—whether used for descriptive or inferential analysis—should be clearly listed. Where appropriate, the appendix or online repository should provide a more detailed (prespecified) statistical analysis plan, ideally covering all primary and secondary outcomes alongside explanatory variables, including prognostic, predictive, confounding variables and effect modifiers. This includes documentation of variable selection procedures, categorisation strategies, modelling steps and assumptions. When drafting results, researchers should report and justify deviations—especially for subgroup, stratified or sensitivity analyses, which are considered exploratory if not prespecified. The examples well describe the type of dependent and explanatory variables, type of models used and the completeness of data.

**Table:** 

17b Methods: data analysis—explain how the sample size was determined.
Examples 1. “We assumed that preventable adverse events would occur in approximately 5% of the admissions, and calculated that a sample of 2000 admissions would be sufficient to estimate the prevalence of preventable adverse events with a 95% CI of +1%. Twenty-eight acute care hospitals were selected to represent the 72 hospitals in Sweden. All large hospitals (> 38 000 admissions), and a random selection of medium (>14 500–38 000 000 admissions) and small (1800–14 500 admissions) hospitals, were included. The selection was representative for the regional distribution and size of hospitals in Sweden. A random selection of medical records, which was representative for hospital size distribution, was performed with the aid of the Swedish National Patient Register. Oversampling was carried out with the expectation that 10% of the medical records would not be available or would be incomplete.”^66^ 2. “We conducted a retrospective surveillance study of randomly selected pediatric inpatient records from 16 teaching and nonteaching hospitals. (…). Each participating hospital randomly selected 10 admissions (lasting≥24 hours) in each quarter from January 2007 through December 2012 (240 records per hospital). (…). Projecting 25 AEs per 100 admissions based on previous studies, we anticipated having 80% power to detect temporal changes in AEs equivalent to a 25% relative reduction in AEs from 2007 to 2012, at a 2-sided 0.05 significance level.”^78^
Explanation Especially when causal inferences are implied, explaining how the sample size was determined ensures the findings are statistically sound, methodologically credible and interpretable—key components for drawing valid and trustworthy conclusions. Sample size calculations conducted a-priori may also be reported for studies not aiming to make causal inferences. If the sample size is based on pragmatic considerations rather than formal calculation, it is advisable for researchers to acknowledge this in the Methods section and outline relevant limitations in the Discussion.

**Table:** 

17c Methods: data analysis—specify the unit(s) used for any presentation of event rates (eg, number of events, number of events per patient, proportion of visits with one or more events, number of events per visit/ hospitalisation, events per 100 visits/ hospitalisations or per 1000 patient days).
Example “Three measures of the rate of AEs were used, including AEs per 1000 patient days, AEs per 100 admissions, and the percentage of entries with at least 1 AE.”^97^
Explanation Specifying units when presenting event rates is crucial for clarity, accurate interpretation and meaningful comparison across studies. Without standardised units, data can be misleading, difficult to replicate and unreliable for analysis or decision-making, potentially resulting in incorrect conclusions or actions. Reporting multiple measures can facilitate comparisons with other studies. In the example, the suggested measures from the GTT are presented.

**Table:** 

17d Methods: data analysis—indicate whether any scales used to assess causation or preventability were collapsed for analysis.
Examples 1. “The assessment of causation was performed using a scale from 1 to 6. At ratings of at least 4 (i.e. more than 50% likelihood), unintended injuries or complications that fulfilled criteria 2 were classified as adverse events.”^66^ 2. “A similar 6-point scale was used to judge the preventability of the AE. A score of four or more meant that the AE was deemed to be preventable.”^81^ 3. “An injury or complication was identified as an AE if it was associated with death, disability at discharge or prolonged hospital stay and received a causation rating of at least 4 (ie, rated as having more than a 50% likelihood of being caused by health care management).”^98^
Explanation Collapsing scales used to assess causation or preventability is a form of data transformation that can reduce nuance and impact interpretation, statistical validity and reproducibility. Clearly reporting whether and how scales were collapsed, as shown in the examples, ensures transparency, prevents misinterpretation and allows others to accurately assess and replicate the study’s findings. Researchers should avoid collapsing multi-point preventability, causation or severity scales without clear justification, as doing so can distort reliability and obscure variation. If the primary justification is that this way of collapsing the results has been commonly performed in the past, this should be explained and should be stated as the justification (ie, consistency with prior research), along with some sense for how this might impact findings. In the examples provided, the creation of a binary result from scales that were created to capture a range of information changes meaning.

**Table:** 

17e Methods: data analysis—describe procedures used to assess and control for bias or confounding.
Examples 1. “The exposure variable in the study was the waiting time to surgery. This was defined as the time that had elapsed in hours, from time of arrival at the Casualty Department to actual time of first incision in theatre. Nine patients sustained fractures while inpatients for other medical conditions. Instead of arrival time at the hospital, the time of confirmed diagnosis i.e. time of X-ray was used in these cases. Confounders are factors with a possible causal effect on both the exposure variable and the outcome variables. The confounders identified in this study were age, sex, type of fracture, comorbidities as reflected in the ASA classification score and the presence of cognitive dysfunction. A directed acyclic graph (DAG) diagram was created prior to the study to help identify possible confounders and adjust for these, thereby reducing possible bias (Fig. 1) (…). A logistic regression analysis was carried out to identify risk factors that in combination with waiting time to surgery had the potential to increase the risk for the occurrence of any SAE (serious adverse event). The statistical model included the exposure variable and confounders (…).”^99^ 2. “In a secondary analysis that was adjusted for the number of patients per resident physician as a potential confounder, intervention schedules were no longer associated with an increase in errors.”^100^
Explanation When evaluating associations between exposure variables and an outcome, the description of statistical methods to control confounding and other sources of bias should be provided. Common biases in AE research include reviewer subjectivity, sampling or selection bias and incomplete documentation. Even when study procedures, such as double review, reviewer calibration or validation against external data, aim to reduce these issues, statistical analyses can still be used to assess their remaining impact. In trigger-based AE detection studies, for example, stratification or multivariable analysis may be used to evaluate whether factors such as patient complexity, care setting or documentation quality influence the relationship between trigger activation and confirmed AEs. Along with sensitivity analyses, these approaches help identify how residual confounding, measurement error or reviewer variability may affect observed associations. Such descriptions are essential for ensuring a study’s validity, credibility and to increase transparency. It helps demonstrate methodological rigour, supports accurate interpretation of results, and allows others to critically evaluate, replicate and responsibly apply the findings. Without this information, conclusions may be misleading and lead to inappropriate decisions or applications. Both examples used analytical adjustments to control for confounding.

**Table:** 

18 Results: participants—report the numbers of participants at each stage of the study—those eligible, excluded (with reasons for exclusion), included and analysed, included but not analysed. Consider including a flow diagram to depict the design and patient flow.
Example “The study population consisted of 21,774 patients. We included 2000 patients weighted according to the selection group table (see online supplementary table A2). Two patients were excluded. The first patient had no available medical record, a short primary admission, no readmissions and was unlikely to have sustained an AE. The second patient had a hip fracture treated with internal fixation, with an assumingly faulty registration in the SHAR (Swedish Hip Arthroplasty Register). After exclusion, 1998 patients with a total of 5422 inpatient admissions and outpatient visits in 69 hospitals were reviewed and included in the analysis (figure 1). The study cohort comprised of 667 acute hip fracture patients and 1331 elective patients, and 63% of the patients were women. The hip fracture group comprised more women, had older patients and involved a longer length of stay during the index admission (table 1).”^50^
Explanation Reporting participant numbers at each stage, along with reasons for exclusions and non-analysis, is vital for a correct understanding of the applied design, enhances transparency and supports the assessment of the validity and generalisability of study findings. A flow diagram is a valuable tool to illustrate this process clearly and concisely. Participant-flow diagrams improve auditability, especially in multistage record reviews.

**Table:** 

19 Results: descriptive data—report data on characteristics of study participants (eg, demographic, clinical, social determinants of health).
Example “The table 1 describes the characteristics of the patient populations at each of the participating sites. In general, patients were older adults (median age 74, IQR 61–84) which was similar across hospitals except for Hospital B, whose patients were slightly younger (median age 67, IQR 56–82). There was a relatively equal gender mix across hospitals except at Hospital D where there were more females (60.7%) than males (39.3%). The most common admitting diagnosis was pneumonia (10.6%) at all sites. (…). There was an uneven distribution of chronic illness with patients in hospitals A and E having a greater burden of chronic illness and patients in hospital D having a lower burden.”^101^
Explanation Reporting participant characteristics is essential for assessing the applicability of study findings to other populations, may help to detect sampling errors, assists the interpretation of study findings in context, and illustrates if potential confounders are measured. As with nearly all items in this checklist, complete reporting enhances transparency, supports replication, enables meaningful subgroup analyses and helps address health disparities by highlighting social determinants of health.

**Table:** 

20 Results: study findings—report the findings of study including all outcomes described in the Methods section.
Example “Over the observation period, there were a total of 800 triggers identified (table 2). (…). The AE risk, which is defined as the number of encounters with at least one AE over the total number of encounters observed, varied between hospitals, ranging from 9.9% in Hospital D to 35.8% in Hospital A. (…). The AE rate per 100 patient days also varied between hospitals, ranging from 1.3 in Hospital D to 6.7 in Hospital A. Hospital A also had the highest rate of preventable AEs (5.2 per 100 patient days). (…). The table 3 summarises AE classifications by type and severity. Of all 356 AEs detected, 45% were related to clinical processes or procedures. (…). In terms of severity, four AEs (1.1%) resulted in, were associated with, or potentially led to death and two AEs (0.55%) led to permanent disability. Most AEs (56%) resulted in symptoms only. Generally, the distribution of type and severity of AEs was similar across sites. The table 4 contains examples of AEs by type.”^101^
Explanation Reporting of study findings on all outcomes as specified in study protocols avoids selective outcome reporting bias. Reporting should include point estimates and whenever appropriate, CIs to assist the reader’s interpretation of precision and statistical significance of findings. If multiple statistical tests were performed in the study, the researchers should specify what adjustments for multiplicity were performed. Such complete reporting provides the necessary overview and specific insights with respect to the study aims and research questions and how these have been answered and addressed. Reporting all outcomes described in the Methods section is essential to ensure methodological transparency, support accurate interpretation and maintain scientific and ethical standards.

**Table:** 

21 Discussion: key results—summarise key results with reference to study objectives and discuss the findings in relation to the existing literature.
Examples 1. “In this study, we successfully implemented the prospective AE surveillance system simultaneously on general medicine wards in five different hospitals. We observed variation in the safety event rates across the five hospitals, with one hospital having an increased risk of AEs and potential AEs. (…). During our implementation, we examined a variety of measurement factors, which might influence the variation in the measured rate, these included patient characteristics, observer behaviours and reviewer AE classification rate. Patient factors, including age and the burden of chronic disease among patients were not independently associated with AE risk. However, there was variation in observer behaviour and there was only moderate agreement among reviewers.”^101^ 2. “Specialized management programs, such as an orthopaedic geriatric comanagement service, have been shown to greatly reduce length of stay, morbidity, and mortality. Our study illustrated that the establishment of a geriatric hip fracture service significantly decreased the rate of adverse events by 12% per year as the program matured. A recent study of adverse events using methods and instruments based on the Harvard Medical Practice Study showed that, of 600 geriatric patients with a hip fracture who had been admitted to either an orthopaedic or surgery service, 20% had an adverse event and 67% of those patients required an additional intervention. (…). Many hospital systems use voluntary or sentinel-event reporting systems to track adverse events that occur in the inpatient setting; however, this has been shown to be inaccurate and to often underestimate the number of adverse events. Rutberg *et al* found that only 6.3% of all adverse events detected with the GTT were voluntarily reported when they compared the 2 techniques. The GTT is a readily available, relatively economical tool that is highly sensitive and specific for identifying adverse events. For this reason, the GTT was chosen for our study to estimate the frequency and severity of adverse events.”^102^
Explanation The discussion section first provides a summary of the main findings, places them in context by comparing them with previous work, and outlines future directions. Linking results to study objectives and existing literature helps clarify whether the research questions were answered and highlights the study’s contribution to advancing the knowledge in the field. It helps readers interpret the findings and its potential impact appropriately.

**Table:** 

22 Discussion: strengths and limitations—discuss strengths and limitations of the study including sources of potential bias, and internal and external validity.
Examples 1. “This study also had some limitations. Prospective surveillance is dependent on observer behaviour and though we moved observers between facilities, we only performed one switch per hospital and we only studied five hospitals (one of which was a community hospital). (…). We also had a limited ability to evaluate variability between reviewers, as we only had a small number of reviewers and they mostly performed reviews from their own hospital. This limited our ability to assess consistency of reviewers, though our demonstrations of moderate inter-rater reliability are consistent with prior studies.”^101^ 2. “One of the strengths of this study is its size (…). The study was carried out at a large acute hospital and with the exception of those with pathological and peri-prosthetic fractures, all patients with hip fractures who were admitted consecutively during the time period of the study were included, thereby helping to eliminate any selection bias. Furthermore, to our knowledge, a linear model for the prediction of risk related to time to surgery has not been previously reported. The study directly reflects the normal clinical setting, which would indicate that our data is valid for the general clinical situation. This implies that the external validity of the study may be regarded as good. Previously, only a few studies have examined the correlation between time to surgery and the occurrence of SAEs during the hospital stay in hip-fracture patients. (…). The use of record review could be seen as a limitation, as it is only as good as the documentation available, but to date, this is the best and most reliable method available (…). The medical record review was carried out by only one of the authors (PKP), which could incorporate an observational bias, but this was controlled for by three senior authors reviewing a random sample of 20 medical records (…). (…) there was no loss to follow-up during the study period.”^99^
Explanation Discussing a study’s strengths and limitations—including potential biases and issues of internal and external validity—supports accurate interpretation of the findings. It helps readers evaluate the generalisability of results and provides a balanced view of the study’s contributions.

## CONCLUSIONS

The development of the SESAME reporting guideline represents an important step towards improving the quality, transparency and reproducibility of studies focused on AEs and medical errors in healthcare. Despite decades of research since the landmark ‘To Err is Human’ report,^103^ substantial variability persists in how these studies are designed, conducted and reported. Key methodological elements—such as explicit definitions of foundational constructs, transparency around approaches to determining causation, details about reviewer characteristics and measures of reliability—are often missing. The adoption of SESAME has the potential to significantly enhance the quality of patient safety research. By standardising reporting, the guideline will facilitate more accurate comparisons across studies, improve the credibility of findings among clinicians and policymakers and ultimately support more effective quality improvement initiatives.

The SESAME statement^3^ is registered with the EQUATOR Network^1^ and the checklist will be available from EQUATOR, this journal and at www.sesamestatement.org We encourage funding agencies and journals publishing studies centred around AEs or medical error to endorse and consider requiring submission of a SESAME checklist for relevant studies. This could accelerate its integration into the research ecosystem. The checklist can be used on its own or in association with other standards that focus on specific methodologies. Adherence to the checklist will ensure researchers include the relevant details so their work is understood, transparent and is reproducible. Previously published statements have been instrumental in improving reporting standards, and our hope is that SESAME will achieve a similar effect in studies of AEs and medical error to facilitate continued improvement. Its widespread adoption promises to advance the science of patient safety, inform evidence-based practice and potentially contribute to meaningful reductions in preventable patient harm.

Future work should focus on evaluating the impact of SESAME on the quality of published manuscripts, as well as developing extensions for specific clinical areas or types of harm. In summary, the SESAME guideline addresses a longstanding need for methodological rigour and transparency in studies of AEs and medical error.

**Table:** 

Strengths and innovations
• Our international, multidisciplinary team used a rigorous, consensus-based process—informed by EQUATOR Network standards—to create a comprehensive, 44-item checklist tailored specifically for studies of AEs and medical error. • The SESAME guideline is distinct from existing reporting frameworks (eg, CONSORT, STROBE, PRISMA) that primarily focus on specific methodologies. Instead, SESAME is focused on patient safety studies evaluating patient harm and medical error regardless of study design, addressing noted gaps in the current landscape of research guidelines. • By convening experts from diverse clinical and methodological backgrounds, we ensured that the checklist is relevant across specialties, care settings and geographic regions, while also incorporating perspectives from patient representatives and health disparities researchers. • A major strength of the SESAME development process was its adherence to established methods for guideline creation, including systematic literature review, a modified Delphi consensus rounds and pilot testing. • The use of anonymous, web-based voting mitigated potential biases and allowed for transparent incorporation of feedback from a large, international group. • The decision to avoid prescribing specific definitions—while encouraging researchers to explicitly state their own—acknowledges the ongoing debate in the field and promotes methodological clarity without stifling innovation. • Initial feedback from those who trialled the checklist on a variety of study designs suggests that SESAME is practical and enhances the clarity and completeness of manuscript reporting. This usability is critical for widespread adoption, as overly complex guidelines are less likely to be implemented in real-world research settings.

## COLLABORATORS

The SESAME Statement Development Team: Lee M Adler DO, Andrew Auerbach MD, MPH, MHM, David Bates MD, MSc, Benjamin Brooke MD, PhD, Andrew Carson-Stevens MRGCP, PhD, Christopher R Carpenter MD, MSc, David Classen MD, MS, Ruth Ann Dorrill MPA, Tejal Ghandi MD, MPH, Richard T Griffey, MD, MPH, Peter D Hibbert MD, Dorothy O Klein PhD, Akira Kuriyama MD, MPH, PhD, Edmund Kwok MD, MHA, MSc, Patricia McGaffigan MS, RN CPPS, Kenneth Michelson MD, MPH, Hassan Murad MD, MPH, Sarah Musy PhD, MSc, Maria Panagioti PhD, Anne W S Rutjes PhD, MSc, Ryan M Schneider ACNP, CPPS, CPHQ, Lucy Schulson MD, MPH, Rene Schwendimann PhD, RN, Sue Sheridan, MIM, MBA, DHL, Michael Simon PhD, RN, Hardeep Singh MD, MPH, David C Stockwell MD, Eric Thomas MD, MPH, Maria Unbeck PhD, RN, Almut Winterstein, RPH, PhD.

## CONTRIBUTORS

All authors contributed to the content and review of the manuscript. RTG is the guarantor.

## FUNDING

The authors have not declared a specific grant for this research from any funding agency in the public, commercial or not-for-profit sectors.

## COMPETING INTERESTS

None declared.

## PATIENT CONSENT FOR PUBLICATION

Not applicable.

## ETHICS APPROVAL

Not applicable.

## PROVENANCE AND PEER REVIEW

Not commissioned; externally peer reviewed.

## ORCID iDs

Richard T Griffey https://orcid.org/0000-0002-6279-7380

David C Stockwell https://orcid.org/0000-0001-6074-5731

Peter D Hibbert https://orcid.org/0000-0001-7865-343X

Dorthe O Klein https://orcid.org/0000-0003-0182-9569

Christopher R Carpenter https://orcid.org/0000-0002-2603-7157

Anne W S Rutjes https://orcid.org/0000-0001-9782-779X

Maria Unbeck https://orcid.org/0000-0002-5090-0352

## References

[1] Equator network: enhancing the quality and transparency of health research. Available: https://www.equator-network.org/reporting-guidelines

[2] Carpenter CR Griffey RT Rutjes AWS. The problem with the existing reporting standards for adverse event and medical error research. BMJ Qual Saf 2025;XXX:273–8. doi:10.1136/bmjqs-2024-017491 10.1136/bmjqs-2024-017491 PMC12802381 39933922

[3] Griffey RT Stockwell DC Adler LM. Standard Elements in Studies of Adverse Events and Medical Error: the SESAME statement. BMJ Qual Saf 2026. doi:10.1136/bmjqs-2025-019458 10.1136/bmjqs-2025-019458 41577438

[4] Gattrell WT Logullo P van Zuuren EJ. ACCORD (ACcurate COnsensus Reporting Document): A reporting guideline for consensus methods in biomedicine developed via a modified Delphi. PLoS Med 2024;21:e1004326. doi:10.1371/journal.pmed.1004326 10.1371/journal.pmed.1004326 PMC10805282 38261576

[5] Brennan TA Leape LL Laird NM. Incidence of Adverse Events and Negligence in Hospitalized Patients. N Engl J Med 1991;324:370–6. doi:10.1056/NEJM199102073240604 10.1056/NEJM199102073240604 1987460

[6] Griffin FA Resar RK. IHI global trigger tool for measuring adverse events (second edition). Cambridge, Massachusetts. Institute for Healthcare Improvement; 2009. Available: http://www.ihi.org/resources/Pages/IHIWhitePapers/IHIGlobalTriggerToolWhitePaper.aspx

[7] Hibbert PD Molloy CJ Hooper TD. The application of the Global Trigger Tool: a systematic review. Int J Qual Health Care 2016;28:640–9. doi:10.1093/intqhc/mzw115 10.1093/intqhc/mzw115 27664822

[8] Panagioti M Khan K Keers RN. Prevalence, severity, and nature of preventable patient harm across medical care settings: systematic review and meta-analysis. BMJ 2019;366:l4185. doi:10.1136/bmj.l4185 10.1136/bmj.l4185 PMC6939648 31315828

[9] Schwendimann R Blatter C Dhaini S. The occurrence, types, consequences and preventability of in-hospital adverse events - a scoping review. BMC Health Serv Res 2018;18:521. doi:10.1186/s12913-018-3335-z 10.1186/s12913-018-3335-z PMC6032777 29973258

[10] Nabhan M Elraiyah T Brown DR. What is preventable harm in healthcare? A systematic review of definitions. BMC Health Serv Res 2012;12:128. doi:10.1186/1472-6963-12-128 10.1186/1472-6963-12-128 PMC3405467 22630817

[11] Thomas EJ Studdert DM Runciman WB. A comparison of iatrogenic injury studies in Australia and the USA. I: Context, methods, casemix, population, patient and hospital characteristics. Int J Qual Health Care 2000;12:371–8. doi:10.1093/intqhc/12.5.371 10.1093/intqhc/12.5.371 11079216

[12] Runciman WB Webb RK Helps SC. A comparison of iatrogenic injury studies in Australia and the USA. II: Reviewer behaviour and quality of care. Int J Qual Health Care 2000;12:379–88. doi:10.1093/intqhc/12.5.379 10.1093/intqhc/12.5.379 11079217

[13] Eggenschwiler LC Rutjes AWS Musy SN. Variation in detected adverse events using trigger tools: A systematic review and meta-analysis. PLoS One 2022;17:e0273800. doi:10.1371/journal.pone.0273800 10.1371/journal.pone.0273800 PMC9436152 36048863

[14] Musy SN Ausserhofer D Schwendimann R. Trigger Tool-Based Automated Adverse Event Detection in Electronic Health Records: Systematic Review. J Med Internet Res 2018;20:e198. doi:10.2196/jmir.9901 10.2196/jmir.9901 PMC6000482 29848467

[15] Plasek JM Amato MG Salem A. Adverse Drug Events in Ambulatory Care: A Cross-Sectional Study. Drug Saf 2025;48:363–74. doi:10.1007/s40264-024-01501-w 10.1007/s40264-024-01501-w PMC12147718 39621298

[16] Griffey RT Schneider RM Todorov AA. Near-Miss Events Detected Using the Emergency Department Trigger Tool. J Patient Saf 2023;19:59–66. doi:10.1097/PTS.0000000000001092 10.1097/PTS.0000000000001092 PMC9974611 36715980

[17] Nilsson L Risberg MB Montgomery A. Preventable Adverse Events in Surgical Care in Sweden: A Nationwide Review of Patient Notes. Medicine (Baltimore) 2016;95:e3047. doi:10.1097/MD.0000000000003047 10.1097/MD.0000000000003047 PMC4839907 26986126

[18] Auerbach AD Lee TM Hubbard CC. Diagnostic Errors in Hospitalized Adults Who Died or Were Transferred to Intensive Care. JAMA Intern Med 2024;184:164–73. doi:10.1001/jamainternmed.2023.7347 10.1001/jamainternmed.2023.7347 PMC10775080 38190122

[19] Pottier P Lagisz M Burke S. Title, abstract and keywords: a practical guide to maximize the visibility and impact of academic papers. Proc Biol Sci 2024;291:20241222. doi:10.1098/rspb.2024.1222 10.1098/rspb.2024.1222 PMC11288685 39079668

[20] Stockwell DC Landrigan CP Toomey SL. Racial, Ethnic, and Socioeconomic Disparities in Patient Safety Events for Hospitalized Children. Hosp Pediatr 2019;9:1–5. doi:10.1542/hpeds.2018-0131 10.1542/hpeds.2018-0131 PMC6600809 30509900

[21] Chaudhari PB Banga A. Writing strategies for improving the access of medical literature. World J Exp Med 2023;13:50–8. doi:10.5493/wjem.v13.i3.50 10.5493/wjem.v13.i3.50 PMC10308323 37396881

[22] Charan GS Kalia R Kaur M. How to Write an Effective Scientific Abstract with Keywords and MeSH Terms. Galician med j 2024;31:3. doi:10.21802/e-GMJ2024-A17

[23] Weingart SN Atoria CL Pfister D. Risk Factors for Adverse Events in Patients With Breast, Colorectal, and Lung Cancer. J Patient Saf 2021;17:e701–7. doi:10.1097/PTS.0000000000000474 10.1097/PTS.0000000000000474 PMC6078829 29419566

[24] Hennessy EA Acabchuk RL Arnold PA. Ensuring Prevention Science Research is Synthesis-Ready for Immediate and Lasting Scientific Impact. Prev Sci 2022;23:809–20. doi:10.1007/s11121-021-01279-8 10.1007/s11121-021-01279-8 PMC8776892 34291384

[25] National Library of Medicine. Finding appropriate mesh headings. Available: https://www.nlm.nih.gov/tsd/cataloging/trainingcourses/mesh/mod4_090.html

[26] Rutberg H Borgstedt-Risberg M Gustafson P. Adverse events in orthopedic care identified via the Global Trigger Tool in Sweden - implications on preventable prolonged hospitalizations. Patient Saf Surg 2016;10:23. doi:10.1186/s13037-016-0112-y 10.1186/s13037-016-0112-y PMC5080833 27800019

[27] Stockwell DC Landrigan CP Schuster MA. Using a Pediatric Trigger Tool to Estimate Total Harm Burden Hospital-acquired Conditions Represent. Pediatr Qual Saf 2018;3:e081. doi:10.1097/pq9.0000000000000081 10.1097/pq9.0000000000000081 PMC6132812 30229193

[28] Pettersson PK Sköldenberg O Samuelsson B. The identification of adverse events in hip fracture patients using the Global Trigger Tool: A prospective observational cohort study. Int J Orthop Trauma Nurs 2020;38:100779. doi:10.1016/j.ijotn.2020.100779 10.1016/j.ijotn.2020.100779 32439319

[29] Eldridge N Wang Y Metersky M. Trends in Adverse Event Rates in Hospitalized Patients, 2010-2019. JAMA 2022;328:173–83. doi:10.1001/jama.2022.9600 10.1001/jama.2022.9600 PMC9277501 35819424

[30] Gadde M Penning M. A Computational Adverse Event Detection Matrix. Stud Health Technol Inform 2020;270:118–22. doi:10.3233/SHTI200134 10.3233/SHTI200134 PMC7928019 32570358

[31] Schildmeijer KGI Unbeck M Ekstedt M. Adverse events in patients in home healthcare: a retrospective record review using trigger tool methodology. BMJ Open 2018;8:e019267. doi:10.1136/bmjopen-2017-019267 10.1136/bmjopen-2017-019267 PMC5781156 29301764

[32] Wong BM Dyal S Etchells EE. Application of a trigger tool in near real time to inform quality improvement activities: a prospective study in a general medicine ward. BMJ Qual Saf 2015;24:272–81. doi:10.1136/bmjqs-2014-003432 10.1136/bmjqs-2014-003432 PMC4387453 25749028

[33] Jorro-Barón F Suarez-Anzorena I Burgos-Pratx R. Handoff improvement and adverse event reduction programme implementation in paediatric intensive care units in Argentina: a stepped-wedge trial. BMJ Qual Saf 2021;30:782–91. doi:10.1136/bmjqs-2020-012370 10.1136/bmjqs-2020-012370 33893213

[34] Vandenbroucke JP von Elm E Altman DG. Strengthening the Reporting of Observational Studies in Epidemiology (STROBE): Explanation and elaboration. Int J Surg 2014;12:1500–24. doi:10.1016/j.ijsu.2014.07.014 10.1016/j.ijsu.2014.07.014 25046751

[35] Page MJ McKenzie JE Bossuyt PM. The PRISMA 2020 statement: An updated guideline for reporting systematic reviews. PLoS Med 2021;18:e1003583. doi:10.1371/journal.pmed.1003583 10.1371/journal.pmed.1003583 PMC8007028 33780438

[36] Hess EP Hollander JE Schaffer JT. Shared decision making in patients with low risk chest pain: prospective randomized pragmatic trial. BMJ 2016;355:i6165. doi:10.1136/bmj.i6165 10.1136/bmj.i6165 PMC5152707 27919865

[37] Hodkinson A Tyler N Ashcroft DM. Preventable medication harm across health care settings: a systematic review and meta-analysis. BMC Med 2020;18:313. doi:10.1186/s12916-020-01774-9 10.1186/s12916-020-01774-9 PMC7646069 33153451

[38] Arumugam A Phillips LR Moore A. Patient and public involvement in research: a review of practical resources for young investigators. BMC Rheumatol 2023;7:2. doi:10.1186/s41927-023-00327-w 10.1186/s41927-023-00327-w PMC9996937 36895053

[39] Staley K Elliott J Stewart D. Who should I involve in my research and why? Patients, carers or the public? Res Involv Engagem 2021;7:41. doi:10.1186/s40900-021-00282-1 10.1186/s40900-021-00282-1 PMC8202960 34127074

[40] Classen DC Resar R Griffin F. “Global trigger tool” shows that adverse events in hospitals may be ten times greater than previously measured. Health Aff (Millwood) 2011;30:581–9. doi:10.1377/hlthaff.2011.0190 10.1377/hlthaff.2011.0190 21471476

[41] Dillner P Unbeck M Norman M. Identifying neonatal adverse events in preterm and term infants using a paediatric trigger tool. Acta Paediatr 2023;112:1670–82. doi:10.1111/apa.16814 10.1111/apa.16814 37151117

[42] Kennerly DA Kudyakov R da Graca B. Characterization of adverse events detected in a large health care delivery system using an enhanced global trigger tool over a five-year interval. Health Serv Res 2014;49:1407–25. doi:10.1111/1475-6773.12163 10.1111/1475-6773.12163 PMC4213042 24628436

[43] Sharma AE Yang J Del Rosario JB. What Safety Events Are Reported For Ambulatory Care? Analysis of Incident Reports from a Patient Safety Organization. Jt Comm J Qual Patient Saf 2020. doi:10.1016/j.jcjq.2020.08.010 10.1016/j.jcjq.2020.08.010 PMC7938864 32980254

[44] Sharek PJ Horbar JD Mason W. Adverse Events in the Neonatal Intensive Care Unit: Development, Testing, and Findings of an NICU-Focused Trigger Tool to Identify Harm in North American NICUs. Pediatrics 2006;118:1332–40. doi:10.1542/peds.2006-0565 10.1542/peds.2006-0565 17015521

[45] Marcus SC Hermann RC Cullen SW. Defining Patient Safety Events in Inpatient Psychiatry. J Patient Saf 2021;17:e1452–7. doi:10.1097/PTS.0000000000000520 10.1097/PTS.0000000000000520 PMC6336525 30020194

[46] Sajith SG Fung DSS Chua HC. The Mental Health Trigger Tool: Development and Testing of a Specialized Trigger Tool for Mental Health Settings. J Patient Saf 2021;17:e360–6. doi:10.1097/PTS.0000000000000606 10.1097/PTS.0000000000000606 PMC8132892 31009409

[47] Nafiu OO Mpody C Aina TA. Race, Ethnicity, and Pediatric Postsurgical Mortality: Current Trends and Future Projections. Pediatrics 2024;154:2. doi:10.1542/peds.2024-065906 10.1542/peds.2024-065906 PMC11291965 39069821

[48] Costello WG Zhang L Schnipper J. Post-Discharge Adverse Events Among African American and Caucasian Patients of an Urban Community Hospital. J Racial Ethn Health Disparities 2021;8:439–47. doi:10.1007/s40615-020-00800-z 10.1007/s40615-020-00800-z PMC7744302 32557279

[49] Landrigan CP Parry GJ Bones CB. Temporal Trends in Rates of Patient Harm Resulting from Medical Care. N Engl J Med 2010;363:2124–34. doi:10.1056/NEJMsa1004404 10.1056/NEJMsa1004404 21105794

[50] Magnéli M Unbeck M Rogmark C. Validation of adverse events after hip arthroplasty: a Swedish multi-centre cohort study. BMJ Open 2019;9:e023773. doi:10.1136/bmjopen-2018-023773 10.1136/bmjopen-2018-023773 PMC6429990 30850403

[51] Avery AJ Sheehan C Bell B. Incidence, nature and causes of avoidable significant harm in primary care in England: retrospective case note review. BMJ Qual Saf 2021;30:961–76. doi:10.1136/bmjqs-2020-011405 10.1136/bmjqs-2020-011405 PMC8606464 33172907

[52] Key MN Shaw AR Erickson KI. A Retrospective Analysis of Serious Adverse Events and Deaths in US-Based Lifestyle Clinical Trials for Cognitive Health. medRxiv 2023. doi:10.1101/2023.09.27.23296243 10.1016/j.conctc.2024.101277 PMC10884817 38404652

[53] Kirkendall ES Kloppenborg E Papp J. Measuring adverse events and levels of harm in pediatric inpatients with the Global Trigger Tool. Pediatrics 2012;130:e1206–14. doi:10.1542/peds.2012-0179 10.1542/peds.2012-0179 23045558

[54] O’Leary KJ Devisetty VK Patel AR. Comparison of traditional trigger tool to data warehouse based screening for identifying hospital adverse events. BMJ Qual Saf 2013;22:130–8. doi:10.1136/bmjqs-2012-001102 10.1136/bmjqs-2012-001102 23038408

[55] Hakkarainen KM Andersson Sundell K Petzold M. Methods for assessing the preventability of adverse drug events: a systematic review. Drug Saf 2012;35:105–26. doi:10.2165/11596570-000000000-00000 10.2165/11596570-000000000-00000 22201475

[56] Zegers M de Bruijne MC Wagner C. Design of a retrospective patient record study on the occurrence of adverse events among patients in Dutch hospitals. BMC Health Serv Res 2007;7:27. doi:10.1186/1472-6963-7-27 10.1186/1472-6963-7-27 PMC1810530 17319971

[57] Forster AJ Taljaard M Bennett C. Reliability of the Peer-Review Process for Adverse Event Rating. PLoS ONE 2012;7:e41239. doi:10.1371/journal.pone.0041239 10.1371/journal.pone.0041239 PMC3406022 22844445

[58] Griffey RT Schneider RM Sharp BR. Practical Considerations in Use of Trigger Tool Methodology in the Emergency Department. J Patient Saf 2021;17:e837–42. doi:10.1097/PTS.0000000000000448 10.1097/PTS.0000000000000448 PMC10183108 29200092

[59] Stockwell DC Bisarya H Classen DC. A Trigger Tool to Detect Harm in Pediatric Inpatient Settings. Pediatrics 2015;135:1036–42. doi:10.1542/peds.2014-2152 10.1542/peds.2014-2152 25986015

[60] Hooper TD Hibbert PD Mealing N. CareTrack Kids—part 2. Assessing the appropriateness of the healthcare delivered to Australian children: study protocol for a retrospective medical record review. BMJ Open 2015;5:e007749. doi:10.1136/bmjopen-2015-007749 10.1136/bmjopen-2015-007749 PMC4390725 25854977

[61] Griffey RT Schneider RM Kwok ESH. Multicenter Trial of the Emergency Department Trigger Tool for Adverse Event Detection. Ann Emerg Med 2025;86:625–38. doi:10.1016/j.annemergmed.2025.06.613 10.1016/j.annemergmed.2025.06.613 40778887

[62] Forster AJ O’Rourke K Shojania KG. Combining ratings from multiple physician reviewers helped to overcome the uncertainty associated with adverse event classification. J Clin Epidemiol 2007;60:892–901. doi:10.1016/j.jclinepi.2006.11.019 10.1016/j.jclinepi.2006.11.019 17689805

[63] Schildmeijer K Nilsson L Arestedt K. Assessment of adverse events in medical care: lack of consistency between experienced teams using the global trigger tool. BMJ Qual Saf 2012;21:307–14. doi:10.1136/bmjqs-2011-000279 10.1136/bmjqs-2011-000279 22362917

[64] Griffey RT Schneider RM Sharp BR. Multicenter Test of an Emergency Department Trigger Tool for Detecting Adverse Events. J Patient Saf 2021;17:e843–9. doi:10.1097/PTS.0000000000000516 10.1097/PTS.0000000000000516 PMC6343477 30395000

[65] Landrigan CP Stockwell D Toomey SL. Performance of the Global Assessment of Pediatric Patient Safety (GAPPS) Tool. Pediatrics 2016;137:e20154076. doi:10.1542/peds.2015-4076 10.1542/peds.2015-4076 27221286

[66] Soop M Fryksmark U Köster M. The incidence of adverse events in Swedish hospitals: a retrospective medical record review study. Int J Qual Health Care 2009;21:285–91. doi:10.1093/intqhc/mzp025 10.1093/intqhc/mzp025 PMC2712321 19556405

[67] Ferner RE Aronson JK. Preventability of drug-related harms - part I: a systematic review. Drug Saf 2010;33:985–94. doi:10.2165/11538270-000000000-00000 10.2165/11538270-000000000-00000 20925436

[68] Calder LA Forster A Nelson M. Adverse events among patients registered in high-acuity areas of the emergency department: a prospective cohort study. CJEM 2010;12:421–30. doi:10.1017/s1481803500012574 10.1017/s1481803500012574 20880432

[69] Hofer TP Bernstein SJ DeMonner S. Discussion between reviewers does not improve reliability of peer review of hospital quality. Med Care 2000;38:152–61. doi:10.1097/00005650-200002000-00005 10.1097/00005650-200002000-00005 10659689

[70] Rafter N Hickey A Conroy RM. The Irish National Adverse Events Study (INAES): the frequency and nature of adverse events in Irish hospitals-a retrospective record review study. BMJ Qual Saf 2017;26:111–9. doi:10.1136/bmjqs-2015-004828 10.1136/bmjqs-2015-004828 PMC5284341 26862223

[71] Stockwell DC Kirkendall E Muething SE. Automated adverse event detection collaborative: electronic adverse event identification, classification, and corrective actions across academic pediatric institutions. J Patient Saf 2013;9:203–10. doi:10.1097/PTS.0000000000000055 10.1097/PTS.0000000000000055 24257063

[72] Winterstein AG Sauer BC Hepler CD. Preventable drug-related hospital admissions. Ann Pharmacother 2002;36:1238–48. doi:10.1345/aph.1A225 10.1345/aph.1A225 12086559

[73] Woo SA Cragg A Wickham ME. Methods for evaluating adverse drug event preventability in emergency department patients. BMC Med Res Methodol 2018;18:160. doi:10.1186/s12874-018-0617-4 10.1186/s12874-018-0617-4 PMC6280499 30514232

[74] Classen D Li M Miller S. An Electronic Health Record-Based Real-Time Analytics Program For Patient Safety Surveillance And Improvement. Health Aff (Millwood) 2018;37:1805–12. doi:10.1377/hlthaff.2018.0728 10.1377/hlthaff.2018.0728 30395491

[75] Classen DC Pestotnik SL Evans RS. Computerized surveillance of adverse drug events in hospital patients. Quality and Safety in Health Care 2005;14:221–6. doi:10.1136/qshc.2002.002972 10.1136/qshc.2002.002972 PMC1744018 15933322

[76] Verlaat CW van der Starre C Hazelzet JA. The occurrence of adverse events in low-risk non-survivors in pediatric intensive care patients: an exploratory study. Eur J Pediatr 2018;177:1351–8. doi:10.1007/s00431-018-3194-y 10.1007/s00431-018-3194-y PMC6096770 29946855

[77] Lindblad M Unbeck M Nilsson L. Identifying no-harm incidents in home healthcare: a cohort study using trigger tool methodology. BMC Health Serv Res 2020;20:289. doi:10.1186/s12913-020-05139-z 10.1186/s12913-020-05139-z PMC7137226 32252755

[78] Stockwell DC Landrigan CP Toomey SL. Adverse Events in Hospitalized Pediatric Patients. Pediatrics 2018;142:2. doi:10.1542/peds.2017-3360 10.1542/peds.2017-3360 PMC6317760 30006445

[79] Wilson RM Runciman WB Gibberd RW. The Quality in Australian Health Care Study. Medical Journal of Australia 1995;163:458–71. doi:10.5694/j.1326-5377.1995.tb124691.x 10.5694/j.1326-5377.1995.tb124691.x 7476634

[80] Davis P Lay-Yee R Briant R. Adverse events in New Zealand public hospitals I: occurrence and impact. N Z Med J 2002;115:U271. 12552260

[81] Unbeck M Lindemalm S Nydert P. Validation of triggers and development of a pediatric trigger tool to identify adverse events. BMC Health Serv Res 2014;14:655. doi:10.1186/s12913-014-0655-5 10.1186/s12913-014-0655-5 PMC4300839 25527905

[82] Matlow AG Baker GR Flintoft V. Adverse events among children in Canadian hospitals: the Canadian Paediatric Adverse Events Study. CMAJ 2012;184:E709–18. doi:10.1503/cmaj.112153 10.1503/cmaj.112153 PMC3447037 22847964

[83] de Vries EN Ramrattan MA Smorenburg SM. The incidence and nature of in-hospital adverse events: a systematic review. Qual Saf Health Care 2008;17:216–23. doi:10.1136/qshc.2007.023622 10.1136/qshc.2007.023622 PMC2569153 18519629

[84] (OIG) OotIG. Adverse events in hospitals: methods for identifying events. D.C.2010. Report No.: OEI-06-08-00221. Washington, 2010.

[85] Carson-Stevens A McFadzean IJ Purchase T. Understanding the scale and nature of avoidable healthcare-associated harm for prisoners in England: protocol for a retrospective cross-sectional study. BMJ Open 2024;14:e085607. doi:10.1136/bmjopen-2024-085607 10.1136/bmjopen-2024-085607 PMC11667317 39806709

[86] Mitchell R Faris M Lystad R. Using the WHO International Classification of patient safety framework to identify incident characteristics and contributing factors for medical or surgical complication deaths. Appl Ergon 2020;82:102920. doi:10.1016/j.apergo.2019.102920 10.1016/j.apergo.2019.102920 31437756

[87] National Coordinating Council for Medication Error Reporting and Prevention (NCC MERP) Index for Categorizing Errors. Available: http://www.nccmerp.org/medErrorCatIndex.html [Accessed 8 Jul 2016]. 10.1002/pds.1423 17523185

[88] Schumock GT Thornton JP. Focusing on the preventability of adverse drug reactions. Hosp Pharm 1992;27:538. 10118597

[89] Hayward RA Hofer TP. Estimating hospital deaths due to medical errors: preventability is in the eye of the reviewer. JAMA 2001;286:415–20. doi:10.1001/jama.286.4.415 10.1001/jama.286.4.415 11466119

[90] Zanetti ACB Dias BM Bernardes A. Incidence and preventability of adverse events in adult patients admitted to a Brazilian teaching hospital. PLoS ONE 2021;16:e0249531. doi:10.1371/journal.pone.0249531 10.1371/journal.pone.0249531 PMC8049336 33857137

[91] Hallas J Harvald B Gram LF. Drug related hospital admissions: the role of definitions and intensity of data collection, and the possibility of prevention. J Intern Med 1990;228:83–90. doi:10.1111/j.1365-2796.1990.tb00199.x 10.1111/j.1365-2796.1990.tb00199.x 2394974

[92] Garrett PR Sammer C Nelson A. Developing and implementing a standardized process for global trigger tool application across a large health system. Jt Comm J Qual Patient Saf 2013;39:292–7. doi:10.1016/s1553-7250(13)39041-2 10.1016/s1553-7250(13)39041-2 23888638

[93] Davis P Lay-Yee R Briant R. Adverse events on new zealand public hospitals: principal findings from a national survey. Wellington, New Zealand. report no.: 9914869513502836. Christchurch, New Zealand University of Otago; 2001. Available: https://ndhadeliver.natlib.govt.nz/delivery/DeliveryManagerServlet?dps_pid=IE6024375

[94] Hommel A Magnéli M Samuelsson B. Exploring the incidence and nature of nursing-sensitive orthopaedic adverse events: A multicenter cohort study using Global Trigger Tool. Int J Nurs Stud 2020;102:103473. doi:10.1016/j.ijnurstu.2019.103473 10.1016/j.ijnurstu.2019.103473 31810021

[95] Griffey RT Schneider RM Kocher KE. The emergency department trigger tool: Multicenter trigger query validation. Acad Emerg Med 2024;31:564–75. doi:10.1111/acem.14873 10.1111/acem.14873 PMC12802392 38497320

[96] Broadley T Serpa Neto A Bailey M. Adverse events during and after early mobilisation: A post hoc analysis of the TEAM trial. Aust Crit Care 2025;38:101156. doi:10.1016/j.aucc.2024.101156 10.1016/j.aucc.2024.101156 39826257

[97] Hu Q Wu B Zhan M. Adverse events identified by the global trigger tool at a university hospital: A retrospective medical record review. J Evid Based Med 2019;12:91–7. doi:10.1111/jebm.12329 10.1111/jebm.12329 30511516

[98] Baker GR Norton PG Flintoft V. The Canadian Adverse Events Study: the incidence of adverse events among hospital patients in Canada. Can Med Assoc J 2004;170:1678–86. doi:10.1503/cmaj.1040498 10.1503/cmaj.1040498 PMC408508 15159366

[99] Kelly-Pettersson P Samuelsson B Muren O. Waiting time to surgery is correlated with an increased risk of serious adverse events during hospital stay in patients with hip-fracture: A cohort study. Int J Nurs Stud 2017;69:91–7. doi:10.1016/j.ijnurstu.2017.02.003 10.1016/j.ijnurstu.2017.02.003 28189926

[100] Landrigan CP Rahman SA Sullivan JP. Effect on Patient Safety of a Resident Physician Schedule without 24-Hour Shifts. N Engl J Med 2020;382:2514–23. doi:10.1056/NEJMoa1900669 10.1056/NEJMoa1900669 PMC7405505 32579812

[101] Forster AJ Huang A Lee TC. Study of a multisite prospective adverse event surveillance system. BMJ Qual Saf 2020;29:277–85. doi:10.1136/bmjqs-2018-008664 10.1136/bmjqs-2018-008664 PMC7146931 31270254

[102] Blood TD Deren ME Goodman AD. Assessment of a Geriatric Hip Fracture Program: Analysis of Harmful Adverse Events Using the Global Trigger Tool. J Bone Joint Surg Am 2019;101:704–9. doi:10.2106/JBJS.18.00376 10.2106/JBJS.18.00376 30994588

[103] Kohn LT Corrigan J Donaldson MS. To err is human: building a safer health system. Washington, D.C: National Academy Press, 2000. 25077248

